# Innovative Methodology for Antimicrobial Susceptibility Determination in *Mycoplasma* Biofilms

**DOI:** 10.3390/microorganisms12122650

**Published:** 2024-12-20

**Authors:** B. Tegner Jacobson, Jessica DeWit-Dibbert, Eli T. Selong, McKenna Quirk, Michael Throolin, Chris Corona, Sobha Sonar, LaShae Zanca, Erika R. Schwarz, Diane Bimczok

**Affiliations:** 1 Department of Microbiology and Cell Biology, Montana State University, Bozeman, MT 59718, USA; bryanjacobson@montana.edu (B.T.J.); jessica.dewit@student.montana.edu (J.D.-D.); mckenna.quirk@student.montana.edu (M.Q.); sobha.sonar@student.montana.edu (S.S.); lashae_z@yahoo.com (L.Z.); 2 Department of Chemistry and Biochemistry, Montana State University, Bozeman, MT 59718, USA; eli.selong@student.montana.edu; 3 Department of Mathematical Sciences, Montana State University, Bozeman, MT 59718, USAcjcorona11@gmail.com (C.C.); 4 Montana Veterinary Diagnostic Laboratory, Montana Department of Livestock, Bozeman, MT 59718, USA; erika.schwarz@mt.gov

**Keywords:** antimicrobial resistance, *Mycoplasma bovis*, *Mycoplasma* sp., biofilm formation, flow cytometry, live/dead staining, assay development

## Abstract

*Mycoplasma* spp. are facultative pathogens that contribute to the pathogenesis of multiple bovine diseases, including the bovine respiratory disease complex, and have been shown to form biofilms. Biofilm formation is associated with increased antibiotic resistance in many organisms, but accurate determination of antimicrobial susceptibility in biofilms is challenging. In *Mycoplasma* spp., antimicrobial susceptibility is routinely determined using metabolic pH-dependent color change. However, biofilm formation can lead to reduced metabolism, making interpretation of metabolic readouts difficult. Therefore, we developed and optimized a new flow cytometry-based method for antimicrobial susceptibility testing in biofilm-forming *Mycoplasma*, termed the live/dead antimicrobial susceptibility test (LD-AST). The LD-AST measures the proportion of live bacteria upon exposure to antibiotics, works robustly with both planktonic and biofilm cultures, and enables the determination of the minimum bactericidal concentration (MBC) for a given antibiotic. We used two strains of *Mycoplasma bovis* (Donetta PG45 and Madison) and two clinical *Mycoplasma bovoculi* isolates (MVDL1 and MVDL2) to determine the impact of biofilm growth on antimicrobial susceptibility for gentamicin, enrofloxacin, or tetracycline. All *Mycoplasma* strains were susceptible to all antibiotics when cultured as planktonic cells, with MBCs in the expected range. However, three out of four strains (Donetta PG45, MVDL1, and MVDL2) were completely resistant to all three antibiotics when newly adhered biofilms were analyzed, whereas *M. bovis* Madison gave variable results. For mature biofilms that were cultured for 4–5 days before antibiotic exposure, results also were variable, with some strains showing an increased resistance with certain antibiotics and a decreased resistance with others. Overall, these results are consistent with earlier reports that biofilms can exhibit increased antimicrobial resistance.

## 1. Introduction

*Mycoplasma* spp. are common facultative pathogens in cattle and other livestock species, where they contribute to significant diseases of the respiratory tract and of other organ systems. In domestic cattle, *Mycoplasma bovis* is considered a key pathogen that significantly contributes to the bovine respiratory disease complex, which causes significant economic losses to the cattle industry globally [1,2]. *M. bovis* is well established as an important cause of pneumonia, mastitis, and arthritis, but has also been associated with ocular infections, otitis media, meningitis, endocarditis, and reproductive disorders [3,4,5,6,7,8]. Clinical signs among cattle infected with *M. bovis* are often inconsistent and unspecific, making infections difficult to identify and control [3,4]. Importantly, studies have shown that *M. bovis* has developed increased levels of resistance to a wide range of antimicrobial classes, including aminocyclitols (spectinomycin), fluoroquinolones (enrofloxacin), lincosamides (clindamycin and lincomycin), macrolides (erythromycin, gamithromycin, tilmicosin, tulathromycin, and tylosin), pleuromutilins (tiamulin), and tetracyclines (oxytetracycline). [9,10,11,12,13,14,15,16]. A second species, *Mycoplasma bovovuli,* is a frequent isolate of the bovine eye [17] and is associated with both bovine conjunctivitis and keratoconjunctivitis [18,19,20,21]. While conjunctivitis has been observed in herds infected with *M. bovoculi* alone [22], *M. bovovuli* has been found to enhance keratoconjunctivitis caused by *Moraxella bovis* [23,24]. Notably, related *Mycoplasma* species have been shown to contribute to similar respiratory diseases in pigs (*Mycoplasma hyopneumoniae*), sheep (*Mycoplasma ovipneumoniae*), and goats (*Mycoplasma ovipneumoniae* and *Mycoplasma arginini*) [25,26,27] and to conjunctivitis in pigs (*Mycoplasma hyorhinitis*), birds (*Mycoplasma gallisepticum*), and cats (*Mycoplasma felis*) [28,29,30].

Antibiotic susceptibility testing (AST) of bacterial isolates is commonly performed in vitro, with results expressed either as the minimum inhibitory concentration (MIC) or the minimum bactericidal concentration (MBC) [31]. The first metric, MIC, is the antibiotic concentration where the growth of the organism is inhibited. The inhibition of an organism’s growth can have clinical relevance, as slowing the spread of the infection can allow for the host immune response to clear the infection. The second metric, MBC, is commonly defined as the minimum concentration of an antibiotic that can kill 99.9% of the viable organisms [31]. The bactericidal effect of the antibiotic can also be interesting clinically, because rather than just inhibiting growth, this can give insight into whether the antibiotic alone can kill the infecting organism. These two metrics can also allow for the comparison between organisms and isolates in vitro, to determine the relative differences in susceptibility to treatment.

There is a need for improved diagnostic methods for *Mycoplasma* spp., specifically in regard to *Mycoplasma* biofilms. To date, there has only been limited research into the effect that biofilm formation has on both the pathogenicity and the antimicrobial resistance of *Mycoplasma* spp. The current method for MIC determination recommended by the International Research Program on Comparative Mycoplasmology (IRPCM) for antimicrobial susceptibility testing (AST) of veterinary *Mycoplasma* spp. utilizes a metabolic assay, termed color change assay, that is based on pH-dependent color change of culture media in a microtiter plate over the course of 7–14 d [32]. Some *Mycoplasma* spp. can produce acid as they metabolize nutrients and grow. When an antibiotic inhibits growth, no acid production and hence no color change occurs in the culture. However, the color change assay can be difficult to interpret, due to the variability of acid production between different *Mycoplasma* strains and under different culture conditions. In particular, metabolic differences between laboratory-adapted *Mycoplasma* strains and clinical isolates can result in variations in acid production [33]. While all planktonic bovine *Mycoplasma* spp. analyzed in our laboratory caused a color change that correlated with growth, others have reported that some *Mycoplasma* spp. do not acidify their media [34]. Previously developed assays to determine the MBC for *Mycoplasma* spp. have been based on the growth rate of the organism or time-kill assays. These methods also rely on a color change by the pH indicator in broth media [35,36,37] or on colony counts on plates [38], which is not always feasible depending on the *Mycoplasma* spp. These issues with the current methods for both MIC and MBC determination point to a need for alternative AST methods.

Biofilm formation is a defense strategy employed by a variety of microorganisms, where the adherence of the cells to a surface and the formation of an extracellular polymeric matrix (EPM) commonly results in both immune evasion and antibiotic resistance [39,40,41]. The increased resistance of biofilms to normal defense mechanisms employed by the host may enable chronic colonization, leading to persistent carrier animals that pose a significant transmission risk to other animals in the population [42,43,44]. In other bacterial species, biofilm formation has been associated with increased antibiotic resistance and negative outcomes in livestock health [45,46]. Biofilm formation by *M. bovis* [43,47] and by other *Mycoplasma* spp. [43,48,49] has been documented in previous studies. However, a recent review by Andrés-Lasheras et al. identified significant knowledge gaps in livestock biofilm research [50]. Specifically, effects of *Mycoplasma* biofilm formation on antimicrobial resistance needs to be addressed due to their potential implications on treatment decisions.

One common feature of biofilms, including those formed by other *Mycoplasma* spp., is a significantly reduced bacterial metabolism, as some bacteria in the biofilm structure enter a dormant metabolic state [51,52,53]. This metabolic dormancy could also lead to a decrease in acid production by *M. bovis,* which could skew the results of the metabolic-based AST method. Due to potential differences in metabolic state and antibiotic susceptibility between planktonic bacteria and biofilm-forming bacteria, antibiotic concentrations may need to be adapted to effectively treat bacterial biofilms. The potential inaccuracy of using the color change assay [32] as well as the rising need for susceptibility information necessitates new approaches to the development of sensitive and rapid AST methods to compare both planktonic *Mycoplasma* spp. as well as *Mycoplasma* biofilms. Therefore, as there are no currently published biofilm-specific methods to address antibiotic susceptibilities for *Mycoplasma* biofilms, we have developed a new method of antimicrobial susceptibility testing. This method, termed the live/dead AST (LD-AST), is based on the quantification of live and dead bacteria in disaggregated biofilms by flow cytometry.

Due to the lack of a cell wall, *Mycoplasma* spp. have an inherent resistance to common antibiotics such as beta-lactams and glycopeptides [54]. Antibiotics utilizing different mechanisms such as inhibiting protein synthesis (tetracyclines and aminoglycosides), or DNA synthesis (fluoroquinolones) have been used to successfully treat *Mycoplasma* infections [54,55]. Therefore, we have selected the antibiotics tetracycline, gentamicin (aminoglycoside), and enrofloxacin (fluoroquinolone), which have been approved for use in clinical practice to treat both bovine respiratory infections (tetracycline and enrofloxacin) and bovine conjunctivitis (gentamicin) [56,57,58,59] to validate the new LD-LST.

## 2. Materials and Methods

### 2.1. Growth Media

Broth media consisted of *Mycoplasma* PPLO Broth Base (19.5 mg/mL) (Becton Dickinson, Franklin Lakes, NJ, USA) supplemented with L-cysteine hydrochloride monohydrate (8.7 × 10^−2^ mg/mL) (Thermo Fisher Scientific, Waltham, MA, USA), beta-nicotinamide adenine dinucleotide (NAD) (8.7 × 10^−2^ mg/mL) (Thermo Fisher Scientific), dextrose (2.6 mg/mL) (Thermo Fisher Scientific), thallium acetate (0.43 mg/mL) (Acros Organics, Fair Lawn, NJ, USA), porcine serum (6.5%) (Quad Five, Rygate, MT, USA), horse serum (6.5%) (Quad Five), and phenol red (2.1 × 10^−2^ mg/mL) (Sigma Aldrich, St. Louis, MO, USA) as a pH indicator.

### 2.2. Bacterial Isolates

*Mycoplasma* isolates (Table 1) were obtained from either the American Type Culture Collection (ATCC, Manassas, VA, USA) or the Montana Veterinary Diagnostic Laboratory (MVDL, Bozeman, MT, USA). The *M. bovoculi* isolates were identified by whole genome sequencing with Illumina MiSeq (Wyoming State Veterinary Laboratory, Laramie, WY, USA). The *M. bovis* PG45 isolate (ATCC, 25523) was used as the reference strain and as a baseline for method development. The resulting optimized methods were then applied to the additional isolates with minimal adjustments.

### 2.3. Culture Methods

Each strain was grown for 2 days (d) in the growth media described above at 37 °C and 5% CO_2_, which were considered the standard conditions for incubation. The culture was incubated in either a polystyrene 96-well or 6-well plate (Table S1). *Mycoplasma* spp. typically do not adhere well to polystyrene, so these were considered planktonic cultures. To stimulate formation of biofilms, the *Mycoplasma* spp. were incubated in a 96-well glass-bottom plate (Cellvis, Product Number: P96-1.5H-N, Mountain View, CA, USA) under the same conditions as above.

### 2.4. Biofilm Image Analysis

*Mycoplasma* biofilm growth over time was determined by inoculating 200 µL of culture at a concentration of 1 × 10^4^ cells/mL into a 96-well glass-bottom plate. The plate was incubated for 6 d under standard conditions. Brightfield images of each well were taken daily using a Keyence BZ-X 810 microscope (Keyence, Itasca, IL, USA). Images were then analyzed using FIJI software (v2.14.0) [60] to assess structure size and confluency of adhered cells. To measure structure size, the images were set to scale in the FIJI software and manually measured using the measurement tool. For confluency analysis of the biofilm, images were analyzed with FIJI software (v2.14.0) [60] using methods adapted from Busschots et al. with some slight modifications. Briefly, the rolling ball radius was reduced until only the background was removed, the threshold was reduced so that only the biofilm was selected, and instead of counting cells, only the area fraction was used [61].

### 2.5. Acid Production Assay

To assess acid production by *Mycoplasma* biofilms, new broth media were placed on the mature biofilms and incubated at standard conditions for 2 d. This was compared to planktonic cultures that were incubated at standard conditions for 2 d. These wells were then imaged to assess visible color change. Acid production by *M. bovis* PG45 was further investigated over a total of 6 d by performing the following experiment: A standard curve of pH values was created to estimate the pH in each well using a SpectraMax ABS Plus plate reader (Molecular Devices, San Jose, CA, USA). The samples and the standard curve were read at 620 nm and 700 nm, and the pH of the sample was calculated based on the standard curve. Each well was then examined for visual signs of contamination or debris, and any wells that were obviously contaminated or contained debris were excluded from the data set.

### 2.6. Quantification of Cells with Flow Cytometry

The ZE5™ Cytometer (Bio-Rad, Hercules, CA, USA) was used for cell quantification of cells, so that standardized concentrations of inoculum could be used. Briefly, 10 µL of ~4 µm Countbright Plus Absolute Counting Beads (Reference Number: C36995, Invitrogen, Waltham, MA, USA) were added to 200 µL of FACS buffer and 100 µL of *Myoplasma* sp. sample. FACS buffer was made using phosphate buffered saline (PBS) (Cytiva, Logan, UT, USA), FBS (2%) (SeraPrime, Fort Collins, CO, USA), and sodium azide (0.1%) (Aqua Solutions Inc., Deer Park, TX, USA). One thousand bead events were collected and then analyzed with the Everest analysis software (v3.2.12.0, Bio-Rad) [62]. The overall concentration for the sample was then calculated with the equation below.

$$Absolute\;count\;\left(\frac{cells}{µL}\right)=\;\frac{\left(Cell\;count\times \;Counting\;beads\;volume\;[µL]\right)}{\left(Counting\;beads\;count\times Cell\;volume\;[µL]\right)}\times Counting\;beads\;concentration\;\left(\frac{beads}{µL}\right)$$

### 2.7. Biofilm Disruption

To analyze *M. bovis* PG45 biofilms using imaging cytometry, the media were removed from the mature biofilms and replaced with 0.2 µm filtered, degassed PBS (Cytiva). The biofilms were then scraped from the bottom of a glass-bottom well with a mini cell scraper (United Biosystems, Herndon, VA, USA) and exposed to either 6 min sonication, 10 min sonication, or Tween 20 (Fisher Scientific) treatment. After scraping, the biofilm suspension was transferred to 1.5 mL microcentrifuge tube (Fisherbrand, Pittsburgh, PA, USA). Using a protocol modified from Totten et al. [63], sonication samples were treated for either 6 min or 10 min at 45 kHz (VWR Scientific, Aquasonic Model 550T, Radnor, PA, USA). Briefly, *Mycoplasma* cultures were sonicated for a longer period using a higher frequency sonicating water bath than was performed by the previous authors. Biofilm disruption with 0.01% Tween 20 was performed for 15 min at room temperature, following a protocol modified from Alsharif and Godfrey [64]. Briefly, Tween 20 was substituted as an alternative for Pluronic F-68 as suggested by the authors. Samples were then stained with SYTO9 (Invitrogen) at a final concentration of 1.36 µM for 15 min at 4 °C and analyzed by imaging cytometry.

### 2.8. Imaging Cytometry Analysis

Imaging cytometry was performed with the Cytek Amnis Imagestream^X^ MkII Imaging flow cytometer (Cytek, Fremont, CA, USA). The particle images were obtained with the 20 × objective so that the field would capture the widest range of particle sizes. Due to the lower limit of size measurement capabilities of the device, the particles were compared by utilizing relative fluorescent units (RFU) following manufacturer recommendations for small particle analysis. Each sample was run on the instrument for 2 min and subsequently analyzed with Amnis IDEAS^®^ 6.2 software. For the analysis, first the samples were gated for a 2 min runtime to ensure consistency, and then the samples were gated for focus in the fluorescent channel. After these first steps were completed, a built-in machine learning module for counting the spots on each image was trained to identify images with multiple particles for removal from the dataset. At this point, the in-focus single particle image fluorescent intensity feature for each particle was exported to a *.csv file for analysis with the R statistical software (v4.3.3, R core Team) [65].

### 2.9. Small Particle Analysis with Flow Cytometry

The disruption method that was determined to be successful by imaging cytometry analysis in Section 2.8 was then analyzed via flow cytometry with the ZE5™ Cytometer (Bio-Rad) utilizing the 405 nm small particle laser. Briefly, the *M. bovis* PG45 culture biofilms were sonicated for 10 min, stained with a 1:6000 dilution of SYBR Safe DNA gel stain (Thermo Fisher), and compared to untreated biofilm and planktonic samples. To determine the specific size of the disrupted particles, a Flow Cytometry Sub-micron Particle Size Reference Kit (Invitrogen) was used. The resulting data were then analyzed with FlowJo software (BD, v. 10.10.0).

### 2.10. Live/Dead Percentage Quantification

**Figure 1:**
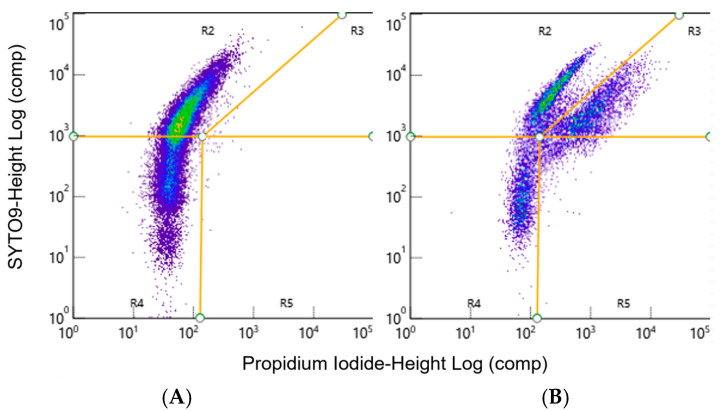
**Representative FACS dot plots of live and dead *M. bovis* samples**. The live/dead stain was visualized as the log intensity of SYTO9 versus the log intensity of the PI (**A**) Live cells were gated as SYTO9-positive, PI-negative cells in gate R2. (**B**) TritonX-100 treatment was used to kill a proportion of the M. bovis to create a positive control. Dead cells are found as SYTO9-positive, PI-positive cells in gate R3. Representative data from one experiment.

To quantify cell death using flow cytometry, 2 d *Mycoplasma* spp. planktonic or biofilms grown in 96-well plates as described above were collected for analysis. The biofilm was collected by removing the medium on top of the biofilm and replacing it with 200 µL of FACS buffer. A mini cell scraper was then used to scrape the bottom of each well and remove the adherent biofilm from the glass-bottom plate. For the planktonic cells 100 µL of the culture was added to 100 µL of FACS buffer and transferred to a capless 1.5 mL microcentrifuge tube (Research Products International, Mt Prospect, IL, USA) and covered with foil (Kirkland Signature, Seattle, WA, USA). Additional disruption treatments were applied after this step as described below. The suspensions of cells and FACS buffer from each tube were individually transferred to the wells of a polystyrene 96-well plate (Greiner, Kremsmünster, Austria) and stained with SYTO9 at a final concentration of 1.36 µM and propidium iodide (PI) at a final concentration of 8 µM, according to methods described by Assunção et al. [66]. A ZE5™ Cytometer (Bio-Rad) was used to analyze 50 µL of each sample. The cell concentration for each sample was then calculated based upon this known volume instead of using the counting beads mentioned above. Data were analyzed using Bio-Rad Everest software (v3.2.12.0) [62] and FlowJo software (v10.10.0). Gates for live and dead cells were applied using the fluorescent intensity of SYTO9 versus PI (Figure 1), as described by Assunção et al. [66]. Separate populations were then visualized for live (Figure 1A) and dead (Figure 1B) bacteria after treating the various *Mycoplasma* spp. with 0.01% Triton X-100 [67] to create a positive control for dead cells (Figure 1B).

### 2.11. Disruption Survival Determination

To ensure that the biofilm disruption treatment which was most successful in reducing the particle size did not result in a decrease in cell viability, 2 d planktonic *M. bovis* PG45 cultures were filtered with a 0.45 µm low protein-binding syringe filter to remove any aggregates. These filtered cells were either left untreated or sonicated for 10 min, as previously described. The cells were then live/dead stained and quantified with both flow cytometry and imaging cytometry.

### 2.12. Cell Adhesion Rate for Newly Adhered Biofilms

To determine the rate at which *M. bovis* PG45 cells adhere to glass, 200 µL of culture were inoculated at a concentration of 2 × 10^4^ cells/mL into the wells of a 96-well glass-bottom plate. After 1, 2.5, or 4 h, the media were removed and replaced with 200 µL of FACS buffer. The cells were then disrupted with a mini cell scraper, transferred to a 1.5 mL microcentrifuge tube (Fisherbrand), and sonicated for 10 min at 45 kHz. These removed cells were then stained with SYTO9, and the cell concentration was determined via flow cytometry.

### 2.13. Broth Microdilutions

Broth microdilutions were made with the growth media mentioned above, but without penicillin and were instead made with either gentamicin (IBISCI, Dubuque, IA, USA), enrofloxacin (Sigma Aldrich, St. Louis, MO, USA), or tetracycline (Sigma Aldrich, St. Louis, MO, USA). Each antibiotic was used with two technical replicates per plate and was diluted with 2-fold serial dilutions into 9 wells. Broth media without antibiotics were used as a growth control. Gentamicin concentrations were 2-fold dilutions from 128–0.5 µg/mL, enrofloxacin from 10–0.039 µg/mL, and tetracycline from 16–0.0625 µg/mL. While both gentamicin and enrofloxacin are considered bactericidal, tetracycline is typically considered bacteriostatic, but bactericidal activity also has been described for certain bacteria [68].

### 2.14. LD-AST Assays for Planktonic Mycoplasma spp., Newly Adhered and Mature Biofilm

For planktonic growth, the microdilutions described above were prepared, and planktonic culture quantified with flow cytometry was inoculated at 200 µL of 1 × 10^4^ cells/mL per well of a polystyrene 96-well plate as recommended by Hannan et al. [32]. The interior wells were inoculated, with the outer edge filled with 1X PBS (Cytiva) to reduce evaporation of the sample wells. Plates were incubated using standard culture conditions for 2 d and then quantified using the LD-AST.

For newly adhered biofilm growth, 200 µL of 2 × 10^4^ cells/mL of planktonic culture was inoculated into the interior wells of a 96-well glass-bottom plate, with the outer edge filled with 1× PBS to reduce evaporation of the sample wells. This plate was then incubated for 1 h at standard conditions so that the adhered cells would fall into the range of 10^3^–10^5^ cells/mL suggested by Hannan et al. [32]. To ensure that the cells were in range, three wells grown for concentration verification were scraped with a mini cell scraper, sonicated for 10 min, and quantified with flow cytometry as described above. The medium on the rest of the newly adhered cells was removed and the prepared antibiotic microdilutions described above were added. Plates were incubated using standard culture conditions for 2 d and then quantified using the LD-AST.

For mature biofilms, 200 µL of 1 × 10^4^ cells/mL of planktonic culture was inoculated into the interior wells of a 96-well glass-bottom plate, with the outer edge filled with 1× PBS to reduce evaporation of the sample wells. This plate was then incubated for 4 d at standard conditions to produce a mature biofilm. The wells were then observed microscopically to ensure consistency of biofilm formation. The media were removed from all sample wells, and newly prepared antibiotic microdilutions as described above were added to the mature biofilms.

### 2.15. Statistical Analysis

All analyses were performed using R Statistical Software (v4.3.3; R Core Team 2024) [65]. Data were processed into data structures with the tidyverse package (v2.0.0) [69]. Linear mixed-models were performed with the nlme package (v3.1.164) [70], generalized linear mixed-models were performed with the lme4 package (v1.1.35.1) [71], and the diagnostic fit was assessed using the ggResidPanel package (v0.3.0) [72]. Mixed model tables were generated with the sjPlot package (v2.8.1.5) [73]. Imaging cytometry particle size data were processed and combined with the purr package (v1.0.2) [74] and stringr package (v1.5.1) [75]. The disruption method survival statistics were performed with the rstatix package (v0.7.2) [76]. All figure plots were made with the ggplot2 package (v3.5.0) [77], ggthemes package (v5.1.0) [78], and ggpubr package (v0.6.0) [79]. For all tests, significance was considered at an α < 0.05.

When determining the maturity of the biofilm described in Section 2.4, we quantified both the largest structure size and the confluence. The effect of incubation times on largest structure size was tested with a separate means mixed model with the day post-inoculation as a fixed effect and the technical replicate as a random effect according to the following:

$$Measurement_{i}∼\delta_{m\left[i\right]}+\beta_{1}X_{DayPostInoculation_{i}}+\epsilon_{i}$$

$$\delta_{m}∼N\left(0,\sigma_{TechnicalReplicate}^{2}\right)$$

$$\epsilon_{i}∼N\left(0,\sigma^{2}\right)$$

where *i* represents the observation level and *m* represents the technical replicate.

The effect of incubation time on biofilm confluence was tested with a separate means mixed model with the day post-inoculation as a fixed effect and the technical replicate as a random effect according to the following:

$$PercentConfluence_{i}∼\delta_{m\left[i\right]}+\beta_{1}X_{DayPostInoculation_{i}}+\epsilon_{i}$$

$$\delta_{m}∼N\left(0,\sigma_{TechnicalReplicate}^{2}\right)$$

$$\epsilon_{i}∼N\left(0,\sigma^{2}\right)$$

where *i* represents the observation level and *m* represents the technical replicate.

The timepoint post-inoculation that was observed to have the largest mean was set as the baseline and compared to all other timepoints post-inoculation.

For the acid production assay described in Section 2.5, a mixed model was applied with an interaction between the inoculation state (uninoculated controls or *M. bovis* PG45) and the antibiotic concentration as the fixed effects and the individual plate variance as a random effect according to the following:

$$pH_{i}∼\delta_{m\left[i\right]}+\beta_{1}X_{InoculationState_{i}}+\epsilon_{i}$$

$$\delta_{m}∼N\left(0,\sigma_{Plate}^{2}\right)$$

$$\epsilon_{i}∼N\left(0,\sigma^{2}\right)$$

where *i* represents the observation level, *m* represents the different 96-well plate blocks, and *j* represents the biofilm percentage.

For the disrupted biofilm data obtained by imaging cytometry described in Section 2.7 and Section 2.8, the percentage of the population ≤1.75 × 10^3^ RFU for each replicate was calculated and a separate means mixed model was performed, with the treatment as a fixed effect and the replicate culture as a random effect according to the following:

$$Percent_{i}∼\delta_{m\left[i\right]}+\beta_{1}X_{Treatment_{i}}+\epsilon_{i}$$

$$\delta_{m}∼N\left(0,\sigma_{ReplicateCulture}^{2}\right)$$

$$\epsilon_{i}∼N\left(0,\sigma^{2}\right)$$

where *i* represents the observation level and *m* represents the biological replicate.

For the small particle 10 min sonication data obtained with the 405 nm small particle laser described in Section 2.9, a generalized linear model was performed, with the treatment as a fixed effect according to the following:

$$log\left({\mathrm{FSC}}_{i}\right)∼\beta_{1}X_{Treatment_{i}}+\epsilon_{i}$$

$$\epsilon_{i}∼IG\left(\mu ,\lambda \right)$$

where *i* represents the observation level.

For the experiments performed, to assess the impact of biofilm disruption on cell viability described in Section 2.11, we wanted to determine whether the treatment resulted in a decrease in live cells. A one-sided Welch’s *t*-test was performed to compare the untreated samples to their paired samples that were sonicated for 10 min. To determine whether there were significant differences between imaging cytometry and flow cytometry, a two-sided Welch’s *t*-test was performed to compare the flow cytometry samples at each treatment to their paired samples analyzed with the imaging cytometer.

For the AST assay described in Section 2.14, we defined the MBC as the lowest dilution that resulted in a significant decrease in live cell percentage that was ≥5%, which we notated as MBC_≥5%_. To determine the MBC_≥5%_, a separate means mixed model was performed for each organism state and antibiotic combination, with concentration as a fixed effect and the biological replicate as a random effect, according to the following:

$$PercentLiveCells_{i}∼\delta_{m\left[i\right]}+\beta_{1}X_{concentration_{i}}+\epsilon_{i}$$

$$\delta_{m}\;∼N(0,\sigma_{ReplicateCulture}^{2})$$

$$\epsilon_{i}∼N\left(0,\sigma^{2}\right)$$

where *i* represents the observation level and *m* represents the biological replicate.

## 3. Results

### 3.1. Biofilm Maturity Determination

**Figure 2:**
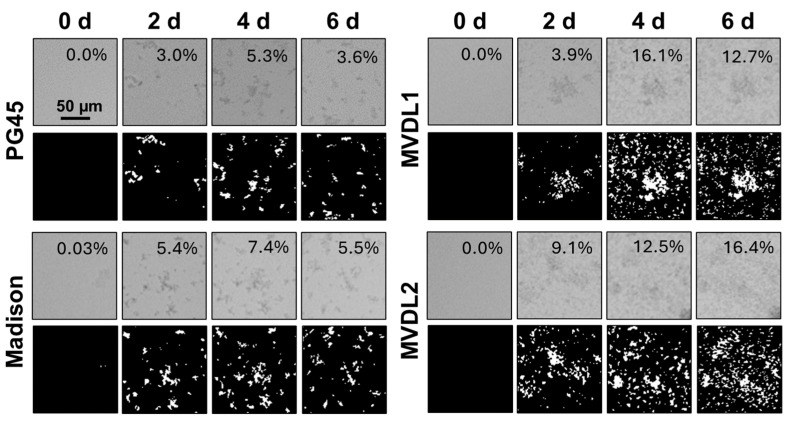
Brightfield images of Mycoplasma biofilm formation on glass-bottom plates over 6 d. Top rows: phase contrast images; bottom rows: thresholded images used to estimate the percent confluence of the biofilm in the field of view. Data are representative of one experiment with 18 technical replicates.

**Figure 3:**
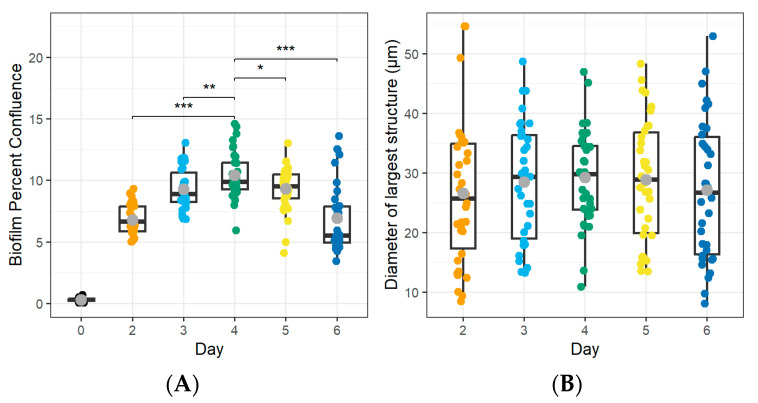
**Maturation of *M. bovis* PG45 biofilms over time**. Biofilm maturity was assessed based on the percent confluence of the cells and the largest structure diameter observed. (**A**) Biofilm confluence peaked at 4 d, which showed strong evidence of a difference (Wald’s Test, *p* < 0.01) compared to all other days post-inoculation. (**B**) The diameter of the largest biofilm structure did not vary across the different days (Wald’s test, *p* > 0.05). The boxplot displays the median and quartiles of the population, the grey point indicates the mean. Representative of two experiments, each with 16–18 technical replicates. * *p* < 0.05, ** *p* < 0.01, *** *p* < 0.001.

First, we confirmed that all four *Mycoplasma* strains produced biofilms following adherence to glass-bottom plates. As shown in Figure 2, adhered bacterial aggregates were visible after 6 days of incubation for all strains, although aggregate sizes varied. Next, we sought to define the point at which the *Mycoplasma* biofilms reached their maximum size and thus could be considered mature based on both the area of the plate covered by the biofilm (confluence) and the size of individual aggregates (structure size). We used digital image analysis to quantify the percent confluence and the diameter of the largest structure after 0–6 d of culture (Figure 3). For *M. bovis* PG45, biofilm confluence peaked after 4 d with a mean of 10.42 ± 1.89% coverage of the culture plate (Figure 3A, Table 2). Biofilm confluence at 4 d showed moderate to strong evidence for a difference (Wald’s test, *p* < 0.05) compared to all other days post-inoculation. Structure size peaked at 6 d with a mean size of 29.50 ± 8.10 µm (Figure 3B, Table 2), with no evidence of differences between any of the time points analyzed (Wald’s test, *p* > 0.05). Based on these results, we considered *M. bovis* PG45 biofilms cultured for 4 d to be mature, since a longer culture did not lead to an increase in biofilm mass. When determining maturity for the additional isolates (Table 2), we found that the *M. bovis* Madison strain had significantly higher confluence (Wald’s Test, *p* < 0.001) at 5 d, with no significant difference in the largest structure size between the other days (Wald’s Test, *p* > 0.05). Therefore, the Madison strain was considered mature after 5 d of incubation. For the *M. bovoculi* MVDL1 isolate, the highest level of confluence was found at 5 d, when it was significantly greater than all other days except 4 d (Wald’s Test, *p* < 0.001), and the largest structure size were present at 3 d, when they were significantly larger than all the other days (Wald’s Test, *p* < 0.001). Therefore, MVDL1 biofilms were considered mature after 4 d. For the *M. bovoculi* MVDL2 isolate, we observed the highest level of confluence at 5 d that was significantly higher than all the other days (Wald’s Test, *p* < 0.001), and we observed the largest structure size at 2 d that was only significantly different than the 3 d and 6 d observations (Wald’s Test, *p* < 0.05). Therefore, MVDL2 biofilms were considered mature after 5 d.

### 3.2. Acid Production

**Figure 4:**
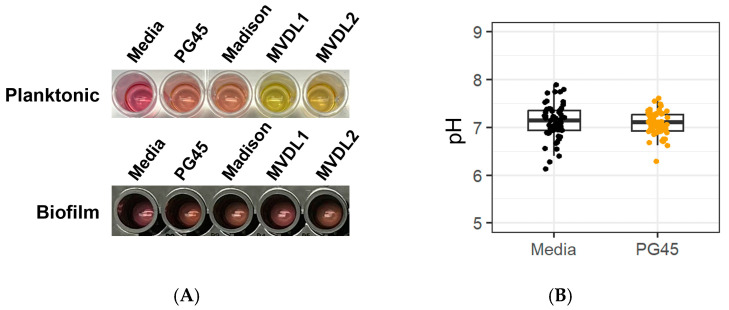
**Standard color change assay is inappropriate for measuring *Mycoplasma* spp. biofilm growth**. (**A**) A color change due to acid production is visible for planktonic cells (top, grown for 2 d on a clear polystyrene plate), but not for mature biofilms (bottom, incubated with new media for 2 d after biofilm formation on a glass-bottom plate with black polystyrene wells). (**B**) Quantification of medium pH for M. bovis PG45 grown as a biofilm for 6 d. No significant difference in pH between the media control and PG45 (Student’s *t*-test, *p* > 0.05). Representative of one experiment with 60 technical replicates.

We first tested the hypothesis that the standard color change assay is not suitable to assess growth and antimicrobial susceptibility of our selected *Mycoplasma* spp. biofilms (Figure 4A). Indeed, while a color change of the media was clearly visible for cultured planktonic *Mycoplasma* spp. compared to the uninoculated control media, no color change was seen in the media of mature *Mycoplasma* spp. biofilms after 2 days. It is interesting to note that variation in acidification was observed between the different organisms. Moreover, additional pH measurements performed with mature *M. bovis* PG45 biofilms over 6 d of incubation showed no evidence (Wald’s test, *p* > 0.05) for a difference in the pH after accounting for the variation by plate (Figure 4B). These data confirm the need for alternative assays to measure antimicrobial effects in *M. bovis* biofilm cultures.

### 3.3. Particle Size Standardization

Having observed that the pH did not change after incubation of a mature biofilm, we developed an innovative flow cytometry-based assay to assess biofilm growth and survival. This assay, termed the live/dead antimicrobial susceptibility test (LD-AST), involves the analysis of disrupted biofilms on a flow cytometer or imaging cytometer. First, we sought to standardize the particle size of the disrupted biofilms as the staining of large cellular aggregates can cause intra-assay variability. *M. bovis* PG45 biofilms were scraped from the plate (untreated control) and then underwent either sonication or detergent treatment with Tween 20 to break up aggregates.

**Figure 5:**
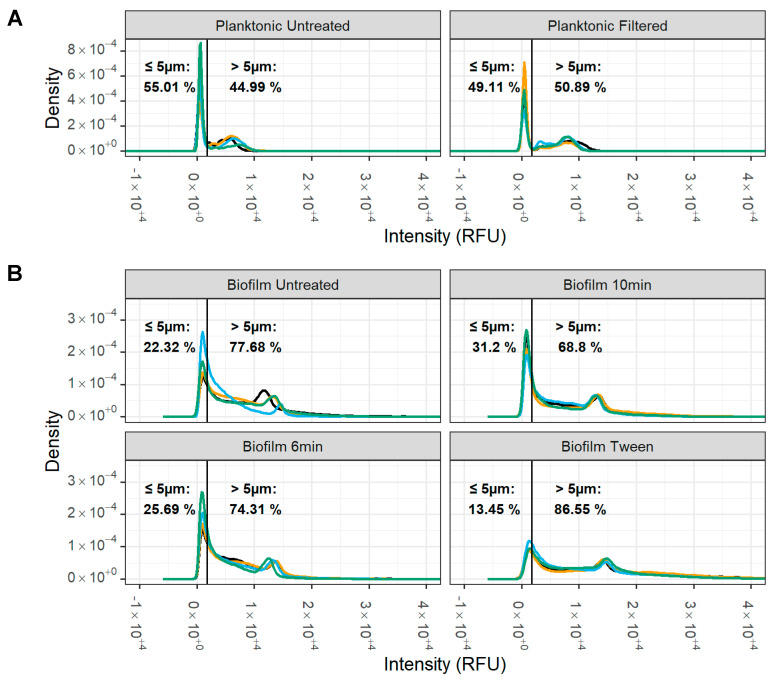
**Impact of biofilm disruption treatment on *M. bovis* particle size**. Particle size of disrupted *M. bovis* biofilms was determined using SYTO9 stained cells with an imaging cytometer. (**A**) Two populations for the planktonic cells were noted, with a natural division observed at 1.75 × 105 RFU, which corresponds with a particle diameter of approximately 5 µm. (**B**) For the disrupted biofilms, the 10 min sonication had strong evidence (Wald’s test, *p* = 0.015) for an increase in small particles compared to the untreated biofilms The percentage of particles above and below 5 µm was calculated for each treatment and compared. Representative of one experiment with two replicate cultures, each with two technical replicates (represented by different colored lines). Each technical replicate had ~5 × 10^4^–8 × 10^4^ particles/biofilm replicate and ~8 × 10^3^–16 × 10^3^ particles/planktonic replicate.

The fluorescence intensity distribution of the planktonic and differentially treated biofilm samples, which correlates with particle size, was measured using imaging cytometry (Figure 5). We observed a bimodal size distribution for planktonic *M. bovis* PG45 (Figure 5A) that we used to define our threshold for large and small particles. The separation occurred at 1.75 × 10^3^ RFU which corresponded to particles with a diameter of 5.05 ± 0.74 µm (A). Based on this definition, planktonic *M. bovis* samples contained 55.01 ± 12.32% of small particles. In contrast, the percentage of small particles in untreated biofilms was 22.32 ± 6.52%. Sonication treatment resulted in a moderate increase in small particles to 25.69 ± 6.33% after 6 min, and a significant increase to 31.20 ± 4.52% (Wald’s test, *p* = 0.015) after 10 min of sonication, whereas Tween 20 treatment resulted a decrease in small particles to 13.45 ± 1.69% (B).

### 3.4. Small Particle Analysis

**Figure 6:**
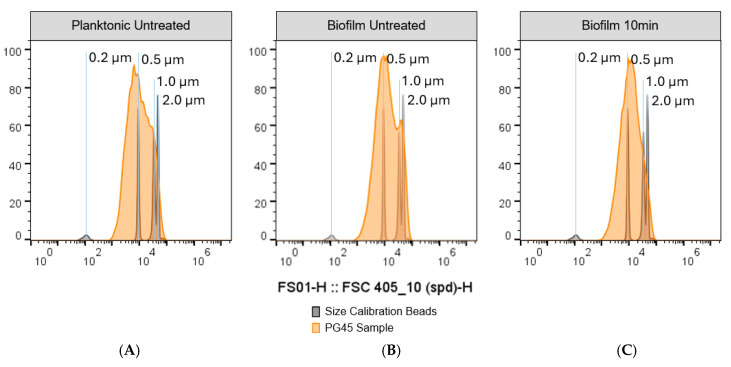
**Impact of 10 min of sonication on the particle size of *M. bovis* PG45 biofilms.** The particle size of disrupted biofilms was further analyzed by observing the forward scatter from a 405 nm small particle laser and comparing it to size calibration beads. (**A**) Planktonic *M. bovis* PG45 culture. (**B**) Untreated *M. bovis* PG45 biofilm. (**C**) *M. bovis* PG45 biofilm after 10 min sonication. Representative graphs were generated by randomly selecting and concatenating 1 × 10^6^ particles from each technical replicate. Representative of one experiment with 4 technical replicates.

Due to the technical limitations of imaging cytometry to quantify both large and small particles at the same time, a second method was used to obtain accurate data on particle size of biofilms treated with 10 min sonication: a flow cytometer equipped with a 405 nm small particle laser and size calibration beads with a range between 0.2 and 2 µm. Single *M. bovis* cells have an expected size range between 0.2 and 1 µm [80,81]. The flow cytometry method revealed that the planktonic *M. bovis* samples had a right skewed distribution with a peak corresponding to approximately 0.5 µm particles (Figure 6A). The untreated biofilm had a bimodal distribution, with the first peak corresponding to ~0.5 µm and the second peak corresponding to 1.0–2.0 µm particles, likely corresponding to aggregated cells (Figure 6B). The 10 min sonicated biofilms had a single peak corresponding to 0.5–1.0 µm particles (Figure 6C).

**Figure 7:**
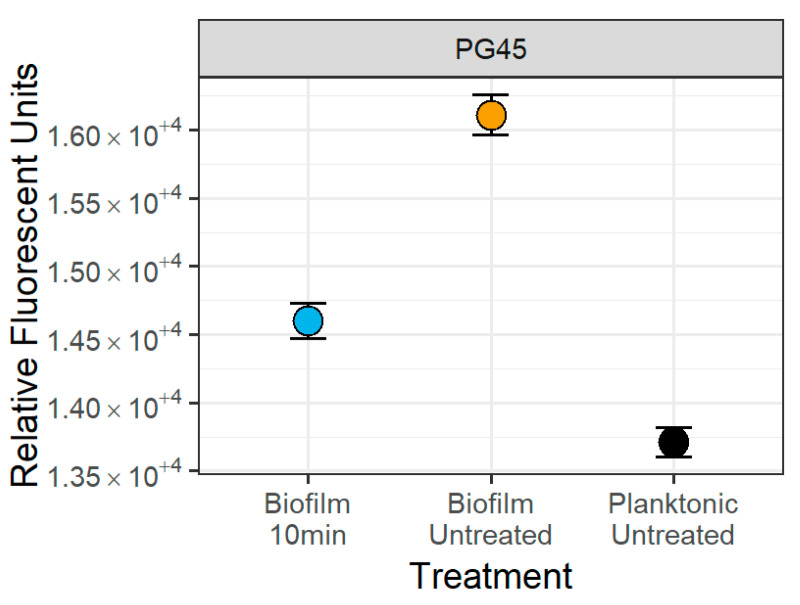
**Impact of 10 min of sonication on the mean fluorescence of the *M. bovis* PG45 particles**. There was a 9.37 ± 0.57% decrease (Wald’s Test, *p* < 0.001) in mean particle size between the untreated biofilm and the 10 min sonicated biofilm. The point is the estimated mean and the 95% confidence interval from the separate means model. Representative of one experiment with four technical replicates.

Consistent with these observations, mean fluorescence values corresponding to size observed were lowest for the planktonic samples, higher for the sonicated biofilms, and highest for the untreated biofilms (Figure 7), with a significant 9.37 ± 0.57% decrease (Wald’s Test, *p* < 0.001) in mean fluorescence after 10 min of sonication compared to the untreated biofilm (Figure 7). Similar findings were obtained with the other *Mycoplasma* isolates.

### 3.5. Disaggregation Method Survival

**Figure 8:**
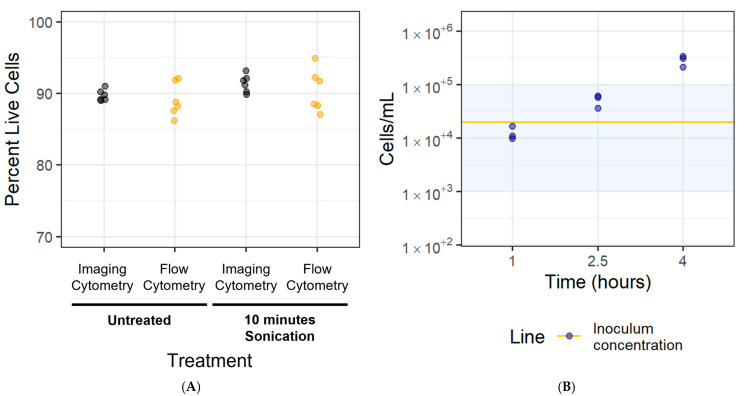
**Analysis of cell viability and density for the LD-AST.** (**A**) The survival of filtered cells exposed to 10 min of sonication and the rate of cell adherence was assessed. Filtered planktonic cells were sonicated for 10 min to determine if the treatment would lead to a decrease in live cells. The imaging and flow cytometry results were compared. Representative of one experiment with six technical replicates. (**B**) Cells inoculated at 2 × 10^4^/mL were imaged at 1, 2.5, and 4 h intervals to determine the rate of adhesion to the glass-bottom plate. The line represents the inoculum concentration while the shaded area represents the 103–105 cells/mL target range. Representative of one experiment with three technical replicates.

Having demonstrated that a 10 min sonication increased the proportion of small particles in the *M. bovis* PG45 biofilms, we next assessed whether the cells survived the sonication treatment. We also sought to ensure that there were no significant differences between measurements obtained using flow cytometry and imaging cytometry. The percentage of live cells (Figure 8A) was generally high, with >90% live bacteria detected with both methods. There was no difference between the untreated and the sonication-treated biofilms (one-sided Welch’s *t*-test, *p* > 0.05), and between measurements obtained using the imaging cytometry and flow (two-sided Welch’s *t*-test, *p* > 0.05).

### 3.6. Cell Adhesion Rate for Newly Adhered Biofilms

To standardize cell counts for the newly adhered biofilm-based assay, the rate at which the cells adhered to the glass-bottom plate was investigated over 1–4 h. Our goal was to achieve a cell concentration of 10^3^–10^5^ cells/mL, which is used in color change MIC assays for *Mycoplasma* [32]. We considered these newly adherent cells as our newly adhered biofilm. *M. bovis* PG45 cells were inoculated at 2 × 10^4^ cells/mL into the wells, incubated for 1–4 h, and then were recovered and analyzed. The mean concentration of adhered cells was 1.26 × 10^4^ ± 3.76 × 10^3^ cells/mL after 1 h of incubation, 5.19 × 10^4^ ± 1.39 × 10^4^ cells/mL after 2.5 h of incubation, and 2.87 × 10^5^ ± 6.89 × 10^4^ cells/mL after 4 h (Figure 8B). Based on these results, we selected an incubation time of 1 h for the newly adhered *M. bovis* PG45 biofilms to achieve a cell concentration in the required 10^3^–10^5^ cells/mL range. When repeating this for the *M. bovis* Madison strain and the *M. bovoculi* MVDL1 and MVDL2 clinical isolates, an incubation time of 30 min was sufficient for reaching the desired cell concentration.

### 3.7. Antimicrobial Susceptibility Testing

**Figure 9:**
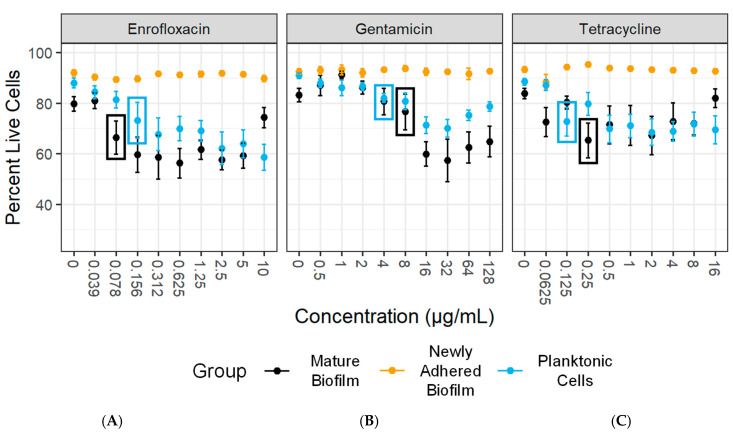
**The LD-AST reveals increased MBCs for *M. bovis* PG45 biofilms compared to planktonic bacteria.** Minimum bactericidal concentration of a decrease in live cells ≥ 5% (MBC ≥ 5%) data showing the percentage of live cells compared to (**A**) enrofloxacin, (**B**) gentamicin, and (**C**) tetracycline concentrations for each of the organism states. The colored boxes correspond to the lowest antibiotic concentration that resulted in a significant drop in the percentage of live cells. The mean and SEM are shown for each group. Representative of three independent experiments with 2 technical replicates.

We next integrated the optimized culture and disruption methods for *Mycoplasma* biofilms into our novel flow cytometry-based LD-AST protocol to measure antibiotic susceptibility of *Mycoplasma* biofilms. The metric defined for MBC_≥5%_ is the lowest concentration that led to a ≥5% decrease in the proportion of live cells. Based on this definition, we observed an MBC_≥5%_ for *M. bovis* PG45 exposed to enrofloxacin of 0.156 µg/mL for the planktonic cells, >10 µg/mL for newly adhered biofilms, and 0.078 µg/mL for mature biofilms (Figure 9A and Table 3). This represents a >64-fold increase in resistance with the newly adhered biofilms compared to the planktonic cells, but a 2-fold decrease in the resistance for the mature biofilm. The MBC_≥5%_ for gentamicin with *M. bovis* PG45 was 4 µg/mL for planktonic cells, >128 µg/mL for newly adhered biofilms, and 8 µg/mL for mature biofilms (Figure 9B and Table 3). This represents a >32-fold and a 2-fold increase in antibiotic resistance for both the newly adhered biofilm and mature biofilms, respectively. The MBC_≥5%_ for *M. bovis* PG45 exposed to tetracycline was 0.125 µg/mL for planktonic cells and increased by >128-fold to >16 µg/mL for newly adhered biofilms, and by 2-fold to 0.25 µg/mL for the mature biofilm (Figure 9C and Table 3). Paradoxically, at the highest concentrations of enrofloxacin (10 µg/mL) and tetracycline (16 µg/mL), the mature biofilm showed an increase in live cells compared to baseline levels, representing resistance to treatment.

When we performed the LD-AST with the additional *Mycoplasma* isolates, we observed that the *M. bovis* Madison strain exposed had no change in susceptibility to enrofloxacin between the planktonic, newly adhered biofilm, and mature biofilm (Table 3). For gentamicin we observed a 2-fold increase in antibiotic resistance between the planktonic cells and newly adhered biofilm, but a 4-fold decrease in resistance with the mature biofilm (Table 3). We also observed that for tetracycline, *M. bovis* Madison had a >2-fold decrease in resistance for both the newly adhered and mature biofilms compared to planktonic cells (Table 3).

For the *M. bovoculi* MVDL1 isolate exposed to enrofloxacin, we observed a >256-fold increase in resistance for the newly adhered biofilms and >8-fold resistance for the mature biofilm compared to the planktonic cells. For gentamicin, we observed that the MVDL1 isolate had >64-fold increase in resistance for the newly adhered biofilm and a >8-fold increase in resistance for the mature biofilm. With tetracycline exposure, the MVDL1 isolate had a >256-fold increase in resistance for the newly adhered biofilm and a >8-fold increase in resistance for the mature biofilm (Table 3). These results show a general trend of large increases in resistance for both the newly adhered biofilms and the mature biofilms for the MVDL1 isolate.

For the *M. bovoculi* MVDL2 isolate exposed to enrofloxacin, we observed a >256-fold increase in resistance for the newly adhered biofilm and no change for the mature biofilm compared to the planktonic cells. For gentamicin, we observed >8-fold increase in resistance with the newly adhered biofilms, and a >4-fold decrease in resistance for the mature biofilms. For tetracycline, we observed a >128-fold increase in resistance for the newly adhered biofilm and >2-fold decrease in resistance for the mature biofilm (Table 3).

### 3.8. Statistical Diagnostics Assessment

The model fit for the separate means mixed models was assessed for both linearity and normality of the residuals and no severe violations were noted for the linearity, but some of the live percentage separate means mixed models had severe violations of normality. Mixed-effect models, however, are robust to violations of normality, with the resulting effect of these violations being a larger uncertainty estimate [82]. The Welch’s *t*-tests that were performed on the disruption survival data were determined to be appropriate as there were no severe violations of the normality assumption.

## 4. Discussion

In this study, we developed an innovative method for antimicrobial susceptibility testing in *Mycoplasma* biofilms. For the culture conditions and *Mycoplasma* strains used here, the pH color change was suitable to monitor growth of planktonic cells (Figure 4A), albeit with variable acidification observed with different *Mycoplasma* strains and isolates. This pH dependent color change, however, was unsuitable for biofilms, since biofilm cultures of bovine *Mycoplasma* spp. did not cause a color change of the culture media upon growth, as we showed. The lack of color change in the media found in our experiment was likely related to either the changes in biofilm metabolism and the metabolic dormancy in the biofilm formation reported for various bacterial species including *Mycoplasma,* or the physical barrier of the EPM altering the diffusion of metabolites [42,51,52,53,83,84,85].

We demonstrated here an innovative AST for *Mycoplasma* biofilms using flow cytometry with live/dead staining with disaggregated *Mycoplasma* biofilms. The application of flow cytometry for the analysis of antimicrobial effects on *Mycoplasma* spp. has been described previously, but only with planktonic organisms [86,87]. Our study sought to standardize methods for utilizing this technique for biofilm-forming *Mycoplasma* spp. Importantly, results for planktonic *M. bovis* PG45 analyzed with our LD-AST (MBC_≥5%_ values) matched previously published values for MIC, at 4 µg/mL for gentamicin [33,88] 0.16 µg/mL for enrofloxacin [88], and 0.125 µg/mL for tetracycline [33]. Interestingly however, no antibiotic concentration was able to reduce the mean percentage of live cells to <45% for any of the tested organisms. This might be due to autoaggregation of the cells, as evidenced from our imaging cytometry data where the population of planktonic cells was 44.99 ± 12.32% larger than 5 µm. Autoaggregation has also been shown to be part of the biofilm formation cycle, and has been previously associated with increases in antibiotic resistance with other organisms, even when not attached to a surface [89,90].

What is interesting about these results generally is that the newly adhered biofilm led to increased resistance in all the tested isolates and antibiotics except for *M. bovis* Madison exposed to enrofloxacin or gentamicin. This phenomenon of biofilms able to withstand increased concentrations of antibiotics has also been termed “tolerance” in several studies [91,92], since decreased susceptibility to antibiotics in biofilms is thought to be caused by the expression of wild-type genes rather than due to genetic mutation or antimicrobial resistance gene acquisition [93]. However, we use the term “resistance”, based on the general definition that resistance is the “development of the ability to withstand the previously destructive effect of a drug by microorganisms” [94] and previous usage of the term “resistance” by other studies to describe this innate antimicrobial resistance by biofilm formation [48,50,95,96]. For newly adhered biofilms, we observed increases in resistance for all three antibiotics in the *M. bovis* PG45, *M. bovoculi* MVDL1, and MVDL2 isolates. For the remaining isolate *M. bovis* Madison, we observed an increase in resistance to gentamicin. These differences in observed susceptibility between the planktonic cells and the biofilm states match previous studies on increased antimicrobial resistance in biofilms formed by other bacteria [49,97].

Data were more variable for mature biofilms. We observed a 2-fold decrease in enrofloxacin resistance for mature biofilms of *M. bovis* PG45, and no change in susceptibility for mature biofilms of *M. bovis* Madison and *M. bovoculi* MVDL2. This might be due to the inhibition of nuclease synthesis by enrofloxacin, as extracellular DNA (eDNA) is important in adherence of other *Mycoplasma* biofilms [98]. The *M. bovis* Madison strain and *M. bovoculi* MVDL2 isolate are also interesting, as both organisms saw a ≥2-fold decrease in susceptibility for both gentamicin and tetracycline. As both organisms took longer to reach peak biofilm maturity (5 d) compared to the other isolates (4 d), and as peak biofilm confluence for *M. bovis* Madison was low (8.99 ± 0.81%), the differential results obtained with the different strains might reflect differences in their ability to form biofilms. For *M. bovis* PG45 and *M. bovoculi* MVDL1, 2-fold and 8-fold increases in resistance, respectively, were observed for both gentamicin and tetracycline MBC_≥5%_ compared to the planktonic cells. The EPM and eDNA of the mature biofilm in these organisms likely provided resistance from gentamicin [97,98,99].

Interestingly, we observed that mature biofilms of *M. bovis* PG45 were completely resistant to the bactericidal effects of the highest concentration of enrofloxacin (10 µg/mL). Similar observations were made for mature biofilms of *M. bovis* PG45, *M. bovis* Madison, and *M. bovoculi* MVDL2 treated with tetracycline, where the highest concentration (16 µg/mL) failed to kill the bacteria, but lower concentrations led to significantly decreased viability. This decrease in efficacy of enrofloxacin in the mature biofilms at the highest concentrations may represent the “paradoxical” or “Eagle effect” [31,100]. The Eagle effect is a phenomenon where higher drug concentrations can result in increased resistance by the organism, while it shows susceptibility to lower drug concentrations [31]. This effect has been seen in both bacterial and fungal biofilms, where increases in antimicrobial concentrations resulted in increased cellular survival in the biofilm [41,101].

Notably, a previous study by McAuliffe et al. [43] investigated *M. bovis* biofilms and found no difference in antimicrobial susceptibility upon biofilm formation. However, the color change assay was used in their study, which cannot accurately determine biofilm growth. Moreover, we showed that the effect of biofilm formation on antibiotic resistance was highly variable and depended on the *Mycoplasma* isolate and the time after biofilm formation, with a strong reduction in antimicrobial susceptibility seen with newly adhered biofilms only. In McAuliffe’s study, biofilms were cultured for 24 h before antibiotics were added [43], whereas we exposed biofilms to antibiotics after 1 h or 4–5 d of growth.

Our results support the hypothesis that *Mycoplasma* biofilms have increased resistance to antibiotics, which may be due to several biofilm-associated mechanisms. First, the EPM can act as a physical barrier to antibiotic diffusion, slowing or preventing interaction of the antibiotics with their cellular targets [102]. Enrofloxacin is a fluoroquinolone antibiotic that inhibits nucleic acid synthesis by disrupting the DNA gyrase and topoisomerase IV enzymes [103]. Resistance to fluoroquinolones has been positively correlated with the bacterial SOS response, which is a series of proteins produced during cellular distress to maintain DNA integrity [104]. This SOS response has been observed as a likely cause of fluoroquinolone resistance in *Mycoplasma gallisepticum* biofilms [105], and genes for the SOS system have been described in *M. bovis* [106]. The second antibiotic we tested, gentamicin, is an aminoglycoside antibiotic that inhibits protein synthesis by causing conformational changes to the 16S ribosomal RNA component of the 30S ribosome subunit, which results in the mistranslation of proteins [99]. Biofilm resistance to aminoglycosides may be partially due to the negative charge of the EPM and the positive charge of the antibiotic, which limits penetration [98]. The eDNA component of the EPM also can contribute to resistance, as even low concentrations in biofilms have been shown to chelate cations and lead to fluoroquinolone resistance [107]. *Mycoplasma* biofilms in other species have both negatively charged EPM as well as eDNA [48,108], which supports a role for both the EPM and the eDNA in the resistance of *M. bovis* biofilms to gentamicin. The third antibiotic that we tested, tetracycline, inhibits protein synthesis by preventing attachment of aminoacyl-tRNA to the ribosomal A acceptor site [109]. In many organisms, expression of efflux pumps that remove antibiotics such as tetracycline from the organism is associated with resistance [98]. Efflux pumps have also been associated with general biofilm formation, as they can contribute to biofilm formation by the secretion of EPM components [110]. Efflux pump-associated genes have also been observed in *M. bovis*, which may help explain our observed resistance to tetracycline by *M. bovis* PG45 [111].

The CLSI states that while the minimal concentration needed to kill 99.9% of the cells is the most common estimation of MBC, this definition is arbitrary and may lack biological relevance [31]. Notably, the MBC_≥5%_ determined with our assay matched the published MIC values obtained by the standard microdilution assay for planktonic *M. bovis* PG45 for all the antibiotics tested, supporting the validity of our approach [33,88].

Glass often allows for the formation of biofilms and *M. bovis* biofilm formation on glass has been previously described [43], as well as for other *Mycoplasma* spp. [108,112]. This informed our choice to use glass-bottom plates in both the newly adhered and mature biofilms. For the newly adhered biofilms, the determination of the cell adherence rate of *M. bovis* was crucial for allowing us to obtain the same starting concentration as our planktonic cells. This approach allowed us to directly compare the new LD-AST to the standard colorimetric assay, as the cell concentration was in the same range when the antibiotics were added.

For the mature biofilms, we wanted to define experimental conditions that were in line with the current understanding of the biofilm life cycle, which involves cell attachment, maturation, and dispersion [50]. Unfortunately, the biofilm formation cycle for *Mycoplasma* spp. is not well described. A few studies have worked to address this in various *Mycoplasma* spp. by characterizing the adhesion, aggregation, and percentage of area covered [113,114], the extracellular matrix composition [108], and gene expression [114]. Even with these studies, a complete understanding of the specific cycle of formation and dispersion is not well understood, and so the application of knowledge from other bacterial species has been applied in this study. In the classical understanding of biofilm formation cycles by other bacteria, the development of a three-dimensional structure is associated with maturity, after which the bacteria disperse, which leads to both the spread of the organism and a decrease in the biofilm biomass [115]. This model matches our observation that the biofilm biomass peaked at 4–5 d, likely representing maturation, and decreased significantly afterwards, possibly reflecting dispersion.

When we investigated the particle size of the planktonic cells, an interesting trend was noted where the filtered cells still had a diameter larger than the 0.45 µm pore size for the syringe filter. The diameter for single *M. bovis* cells is between 0.2–1.0 µm [80,81], and our flow cytometry experiments confirmed that planktonic *M. bovis* was within the 0.5–1.0 µm range. In a study investigating filtration, soft particles were able to deform and translocate across filter pores that were smaller than the particles [116]. As *Mycoplasma* spp. lack a cell wall, the bacteria likely passed through the filter pores by deformation.

We are aware that this study still had some limitations, as we only utilized four *Mycoplasma* isolates, three antibiotics, and a limited number of disruption techniques. Our study also only performed a limited investigation into *Mycoplasma* biofilm maturity and did not investigate any mechanisms of resistance conferred by the formation of the biofilms. These are important factors for the understanding and characterization of both biofilms and resistance by *M. bovis* and should be explored further. Also, we did not validate our AST results with the gold standard color change assay, but instead relied on published MIC values. While these limitations do exist, the overall methodology should remain viable with minimal adaptation for other antibiotics, *M. bovis* and *M. bovoculi* strains, and *Mycoplasma* spp.

In summary, we utilized the LD-AST to directly compare the antibiotic susceptibilities of *M. bovis* biofilms and planktonic cells, with reproducible results. The MBC_≥5%_ we observed for planktonic cells corresponded with the MIC values obtained with the standard microdilution method. We anticipate that the new LD-AST method will allow for further comparison of the resistive effects of biofilm formation by other *Mycoplasma* spp. and will better inform whether antibiotic treatments might be successful in treating infection.

## Figures and Tables

**Table 1:** Characteristics of bovine *Mycoplasma* isolates used in this study.

Mycoplasma Isolate	Source	Isolation Source	Year Isolated
*M. bovis* strain Donetta PG45	American Type Culture Collection (ATCC) 25523	Bovine mastitis	1962
*M. bovis* strain Madison	ATCC 27368	Dairy products	1971
*M. bovoculi* Clinical Isolate MVDL1	Montana Veterinary Diagnostic Lab (MVDL)	Bovine conjunctivitis	2023
*M. bovoculi* Clinical Isolate MVDL2	MVDL	Bovine conjunctivitis	2023

**Table 2:** The peak maturity metrics observed over 6 days of growth.

Mycoplasma Isolate	Mature Biofilm Confluence	Largest Structure Size
PG45	10.42 ± 1.89% (4 days)	29.50 ± 8.10 µm (4 days)
Madison	8.99 ± 0.81% (5 days)	30.40 ± 9.38 µm (2 days)
MVDL1	26.38 ± 4.12% (5 days)	60.48 ± 25.18 µm (3 days)
MVDL2	22.84 ± 2.94% (5 days)	50.09 ± 13.82 µm (2 days)

**Table 3:** MBC_≥5%_ concentration in µg/mL by organism state (planktonic, newly adhered biofilms, mature biofilm).

	Antimicrobial Agent	*Mycoplasma bovis* State		
*Mycoplasma* Isolate		Planktonic	Newly Adhered Biofilm	Mature Biofilm
PG45	Enrofloxacin	0.156	>10 ^1^	0.078
	Gentamicin	4	>128 ^1^	8
	Tetracycline	0.125	>16 ^1^	0.25
Madison	Enrofloxacin	0.078	0.078	0.078
	Gentamicin	8	16	2
	Tetracycline	0.125	<0.0625 ^2^	<0.0625 ^2^
MVDL1	Enrofloxacin	<0.039 ^2^	>10 ^1^	0.312
	Gentamicin	2	>128 ^1^	16
	Tetracycline	<0.0625 ^2^	>16 ^1^	0.5
MVDL2	Enrofloxacin	0.078	>10 ^1^	0.078
	Gentamicin	16	>128 ^1^	4
	Tetracycline	0.125	>16 ^1^	<0.0625 ^2^

^1^ MBC ≥ 5% higher than the concentrations of the tested range, ^2^ MBC > 5% lower than the concentrations of the tested range.

## Data Availability

The data for the experiments described above can be found at: https://doi.org/10.5061/dryad.m905qfv8m.

## Supplementary Materials

The following supporting information can be downloaded at: https://www.mdpi.com/article/10.3390/microorganisms12122650/s1, Table S1: Catalog numbers and manufacturers for the materials used; Table S2: Estimated pH model output; Table S3: Particle disruption model output; Table S4: Small particle (405 nm) model output; Table S5: PG45 maturity structure measurement model; Table S6: PG45 maturity confluence model; Table S7: Madison maturity structure measurement model; Table S8: Madison maturity confluence model; Table S9: MVDL1 maturity structure measurement model; Table S10: MVDL1 maturity confluence model; Table S11: MVDL2 maturity structure measurement model; Table S12: MVDL2 maturity confluence model; Table S13: Mature PG45 percent live model (PLM) for enrofloxacin; Table S14: Mature PG45 PLM for gentamicin; Table S15: Mature PG45 PLM for tetracycline; Table S16: NAB PG45 PLM for enrofloxacin; Table S17: NAB PG45 PLM for gentamicin; Table S18: NAB PG45 PLM for tetracycline; Table S19: Planktonic PG45 PLM for enrofloxacin; Table S20: Planktonic PG45 PLM for gentamicin; Table S21: Planktonic PG45 PLM for tetracycline; Table S22: Mature Madison PLM for enrofloxacin; Table S23: Mature Madison PLM for gentamicin; Table S24: Mature Madison PLM for tetracycline; Table S25: NAB Madison PLM for enrofloxacin; Table S26: NAB Madison PLM for gentamicin; Table S27: NAB Madison PLM for tetracycline; Table S28: Planktonic Madison PLM for enrofloxacin; Table S29: Planktonic Madison PLM for gentamicin; Table S30: Planktonic Madison PLM for tetracycline; Table S31: Mature MVDL1 PLM for enrofloxacin; Table S32: Mature MVDL1 PLM for gentamicin; Table S33: Mature MVDL1 PLM for tetracycline; Table S34: NAB MVDL1 PLM for enrofloxacin; Table S35: NAB MVDL1 PLM for gentamicin; Table S36: NAB MVDL1 PLM for tetracycline; Table S37: Planktonic MVDL1 PLM for enrofloxacin; Table S38: Planktonic MVDL1 PLM for gentamicin; Table S39: Planktonic MVDL1 PLM for tetracycline; Table S40: Mature MVDL2 PLM for enrofloxacin; Table S41: Mature MVDL2 PLM for gentamicin; Table S42: Mature MVDL2 PLM for tetracycline; Table S43: NAB MVDL2 PLM for enrofloxacin; Table S44: NAB MVDL2 PLM for gentamicin; Table S45: NAB MVDL2 PLM for tetracycline; Table S46: Planktonic MVDL2 PLM for enrofloxacin; Table S47: Planktonic MVDL2 PLM for gentamicin; Table S48: Planktonic MVDL2 PLM for tetracycline.
