# Correction: Jacobson et al. Innovative Methodology for Antimicrobial Susceptibility Determination in *Mycoplasma* Biofilms. *Microorganisms* 2024, *12*, 2650

**DOI:** 10.3390/microorganisms13102331

**Published:** 2025-10-10

**Authors:** B. Tegner Jacobson, Jessica DeWit-Dibbert, Eli T. Selong, McKenna Quirk, Michael Throolin, Chris Corona, Sobha Sonar, LaShae Zanca, Erika R. Schwarz, Diane Bimczok

**Affiliations:** 1 Department of Microbiology and Cell Biology, Montana State University, Bozeman, MT 59718, USA; bryanjacobson@montana.edu (B.T.J.); jessica.dewit@student.montana.edu (J.D.-D.); mckenna.quirk@student.montana.edu (M.Q.); sobha.sonar@student.montana.edu (S.S.); lashae_z@yahoo.com (L.Z.); 2 Department of Chemistry and Biochemistry, Montana State University, Bozeman, MT 59718, USA; eli.selong@student.montana.edu; 3 Department of Mathematical Sciences, Montana State University, Bozeman, MT 59718, USA; michael.throolin@utah.edu (M.T.); cjcorona11@gmail.com (C.C.); 4 Montana Veterinary Diagnostic Laboratory, Montana Department of Livestock, Bozeman, MT 59718, USA

In the original publication [1], there was an error in the notation for the model on the bottom of page 8 (Section 2.15: Statistical Analysis), where it reads

$$log\left(FSC_{i}\right)∼\beta_{1}X_{Treatment_{i}}+\epsilon_{i}\;\epsilon_{i}∼IG\left(\mu ,\lambda \right)$$

where *i* represents the observation level.

The correct notation for the model fit by the statistical package we used is as follows:

$$log\left(\mu_{i}\right)=\beta_{0}+\beta_{1}X_{Treatment_{i}}\;FSC_{i}∼IG\left(\mu_{i},\lambda \right)$$

where *i* represents the individual observation, and *IG* refers to the inverse Gaussian distribution.

As this is an issue with the notation of a model in the methods section, it does not affect the results of the study. It does, however, affect the reproducibility of our methods, as the current notation is not equivalent to what was fitted into R.

This correction was approved by the Academic Editor. The original publication has also been updated.
