# Supplementary material for: Innovative Methodology for Antimicrobial Susceptibility Determination in Mycoplasma Biofilms
Source: Microorganisms. 2024 Dec 20;12(12):2650. doi: 10.3390/microorganisms12122650 (PMC11728330; doi:10.3390/microorganisms12122650)
Supplement: Supplementary file 1 [file microorganisms-12-02650-s001.zip › microorganisms-3263527-supplementary.pdf]

Supplementary Table S1. Catalog numbers and manufacturers for materials used.

Product Name	Manufacturer	Catalog Number
BBL Mycoplasma Broth Base	Becton Dickinson Company	REF: 211458
L-Cysteine Hydrochloride	Fisher Scientific	BP376-100
Beta-NAD	Sigma-Aldrich	N3014-1G
Dextrose	Fisher Scientific	D14-500
Thallium Acetate	Acros Organics	420770250
Porcine Serum	Quad Five	26250-084
Horse Serum	Quad Five	DH-07
Phenol Red	Sigma-Aldrich	P3532-5G
Enrofloxacin	Sigma-Aldrich	17849-5G-F
Tetracycline	Sigma-Aldrich	200-593-8
Triton X	Fisher Scientific	BP151-100
SYTO9	Invitrogen	S34854
Gentamicin	IBISCI Scientific	IB 02030
Propidium Iodide	Sigma	P 4170
Tween-20	Fisher Scientific	BP337-500
Countbright Counting Beads	Invitrogen	REF: C36995
Glass Bottom 96-Well Plate	Cellvis	P96-1.5H-N
Polystyrene 96-Well Plate	Celltreat	229195
Polystyrene 6-Well Plate	Celltreat	229105
0.45 uM Syringe Filter	Fisher Scientific	09-719H
Mini Cell Scrapers	United Biosystems	MCS-200
Keyence	Keyence	BZ-X810
Flow Cytometer	Bio-Rad	ZE5 Cell Analyzer
Plate Reader	Molecular Devices	SpectraMax ABS Plus
Bath Sonicator	VWR Scientific	Aquasonic-Model 550T
ImageStream	Amnis	MKII
Phosphate buffered saline	Cytiva	SH30256.02
Sodium Azide	Aqua Solutions, Inc.	26628-22-8
Fetal Bovine Serum	SeraPrime	F31016HI-500
1.5 mL microcentrifuge tubes	Fisherbrand	05-408-129
1.5 mL microcentrifuge tubes (capless)	Research Products International	145657
Greiner 96-well plate	Greiner	655101
Aluminum Foil	Kirkland Signature	RK624
Mini Cell Scrapers	United Biosystems	MCS-200
SYBR Safe DNA Gel Stain	Invitrogen	S33102
Flow Cytometry Sub-Micron Size Reference Kit, Green Fluorescent	Invitrogen	F13839

Supplementary Table S2. Estimated pH model output

	pH		
Predictors	*Estimates*	*CI*	*p*
(Intercept) Uninoculated Media	7.12	7.03 – 7.21	**<0.001**
M. bovis PG45	-0.03	-0.14 – 0.07	0.542
N $_{plate}$	10		
Observations	120		

Supplemetary Table S3. Particle disruption model output

	Percent of population ≤ 1.75 x 10^{3} RFU		
Predictors	*Estimates*	*CI*	*p*
(Intercept)	22.32	15.29 – 29.36	**<0.001**
Treatment [10min Sonication]	8.88	2.05 – 15.71	**0.015**
Treatment [6min Sonication]	3.37	-3.46 – 10.19	0.301
Treatment [Tween]	-8.87	-15.70 – -2.04	**0.016**
N $_{Replicate\ Culture}$	2		
Observations	16		

Supplementary Table S4. Small particle (405 nm laser) modelfor 10 minute sonicated samples

	FSC		
Predictors	*Estimates*	*CI*	*p*
(Intercept) Untreated Biofilm	16109.38	16035.44 – 16184.01	**<0.001**
Treatment [10min sonication]	0.91	0.90 – 0.91	**<0.001**
Treatment [Untreated Planktonic]	0.85	0.84 – 0.86	**<0.001**
Observations	359452		

Supplementary Table S5. *M. bovis* PG45 maturity structure measurement model output

	Diameter of largest structure		
Predictors	*Estimates*	*CI*	*p*
(Intercept) Day 4	27.07	23.78 – 30.36	**<0.001**
Day 2	-2.96	-6.42 – 0.50	0.093
Day 3	-0.74	-3.67 – 2.19	0.617
Day 5	-0.38	-3.31 – 2.54	0.796
Day 6	-2.09	-5.01 – 0.84	0.160
$N_{\text{Technical Replicate}}$	34		
Observations	170		

Supplementary Table S6. *M. bovis* PG45 maturity confluence model output

	Percent confluence		
Predictors	*Estimates*	*CI*	*p*
(Intercept) Day 4	10.37	9.72 – 11.03	**<0.001**
Day 2	-3.75	-4.63 – -2.87	**<0.001**
Day 3	-1.14	-1.98 – -0.29	**0.009**
Day 5	-1.10	-1.94 – -0.25	**0.011**
Day6	-3.49	-4.34 – -2.65	**<0.001**
$N_{\text{Technical Replicate}}$	34		
Observations	170		

Supplementary Table S7. *M. bovis* Madison maturity structure measurement model output

	Diameter of largest structure		
Predictors	*Estimates*	*CI*	*p*
(Intercept) Day 2	30.40	25.91 – 34.90	**<0.001**
Day 5	-2.18	-8.54 – 4.17	0.494
Day 3	-3.57	-9.92 – 2.79	0.265
Day 4	-2.94	-9.30 – 3.41	0.357
Day 6	-6.03	-12.38 – 0.33	0.063
$N_{\text{Technical Replicate}}$	18		
Observations	90		

Supplementary Table S8. *M. bovis* Madison maturity confluence model output

	Percent confluence		
Predictors	*Estimates*	*CI*	*p*
(Intercept) Day 5	8.99	8.68 – 9.30	**<0.001**
Day 2	-2.70	-3.15 – -2.26	**<0.001**
Day 3	-1.63	-1.95 – -1.31	**<0.001**
Day 4	-0.83	-1.15 – -0.50	**<0.001**
Day 6	-5.02	-5.34 – -4.70	**<0.001**
N Technical Replicate	18		
Observations	90		

Supplementary Table S9. *Mycoplasma sp.* MVDL1 maturity structure measurement model output

	Diameter of Largest Structure		
Predictors	*Estimates*	*CI*	*p*
(Intercept) Day 3	60.48	48.32 – 72.63	**<0.001**
Day 2	-8.93	-17.29 – -0.58	**0.036**
Day 4	-10.80	-19.16 – -2.44	**0.012**
Day 5	-14.89	-23.24 – -6.53	**0.001**
Day6	-24.94	-33.30 – -16.58	**<0.001**
N Technical Replicate	18		
Observations	90		

Supplementary Table S10. *Mycoplasma sp.* MVDL1 maturity confluence model output

	Percent confluence		
Predictors	*Estimates*	*CI*	*p*
(Intercept) Day 4	26.01	24.41 – 27.61	**<0.001**
Day 2	-20.20	-21.88 – -18.52	**<0.001**
Day 3	-6.88	-8.56 – -5.19	**<0.001**
Day 5	0.37	-1.31 – 2.05	0.662
Day6	-15.41	-17.09 – -13.72	**<0.001**
N Technical Replicate	18		
Observations	90		

Supplementary Table S11. *Mycoplasma sp.* MVDL2 maturity structure measurement model output

	Diameter of Largest Structure		
Predictors	*Estimates*	*CI*	*p*
(Intercept) Day 2	50.09	43.81 – 56.38	**<0.001**
Day [5]	-8.24	-15.94 – -0.55	**0.036**
Day [3]	-3.18	-10.87 – 4.52	0.413
Day [4]	-6.84	-14.53 – 0.86	0.081
Day [6]	-11.91	-19.61 – -4.21	**0.003**
N Technical Replicate	18		
Observations	90		

Supplementary Table S12. *Mycoplasma sp.* MVDL2 maturity confluence model output

	Percent confluence		
Predictors	*Estimates*	*CI*	*p*
(Intercept) Day 2	50.09	43.81 – 56.38	**<0.001**
Day 5	-8.24	-15.94 – -0.55	**0.036**
Day 3	-3.18	-10.87 – 4.52	0.413
Day 4	-6.84	-14.53 – 0.86	0.081
Day 6	-11.91	-19.61 – -4.21	**0.003**
N Technical Replicate	18		
Observations	90		

Supplementary Table S13. Percent live model output for mature *M. bovis* PG45 biofilms exposed to enrofloxacin.

	Live Percent		
Predictors	*Estimates*	*CI*	*p*
(intercept) concentration [0 μg/mL]	79.74	66.76 – 92.73	**<0.001**
concentration [0.039 μg/mL]	1.29	-11.46 – 14.05	0.839
concentration [0.078 μg/mL]	-13.37	-26.12 – -0.62	**0.040**
concentration [0.156 μg/mL]	-20.15	-32.90 – -7.40	**0.003**
concentration [0.312 μg/mL]	-21.15	-33.90 – -8.39	**0.002**
concentration [0.625 μg/mL]	-23.45	-36.20 – -10.70	**0.001**
concentration [1.35 μg/mL]	-18.14	-30.89 – -5.39	**0.006**
concentration [2.5 μg/mL]	-22.15	-34.91 – -9.40	**0.001**
concentration [5 μg/mL]	-20.59	-33.34 – -7.84	**0.002**
concentration [10 μg/mL]	-5.43	-18.18 – 7.32	0.396
$N_{Replicate\ Culture}$	3		
Observations	60		

Supplementary Table S14. Percent live model output for mature *M. bovis* PG45 biofilms exposed to gentamicin.

	Live Percent		
Predictors	*Estimates*	*CI*	*p*
(intercept) concentration [0 μg/mL]	83.22	71.56 – 94.87	**<0.001**
concentration [0.5 μg/mL]	3.82	-10.09 – 17.73	0.584
concentration [1 μg/mL]	8.00	-5.90 – 21.91	0.253
concentration [2 μg/mL]	2.89	-11.02 – 16.80	0.678
concentration [4 μg/mL]	-2.52	-16.43 – 11.39	0.717
concentration [8 μg/mL]	-6.65	-20.56 – 7.26	0.341
concentration [16 μg/mL]	-23.30	-37.21 – -9.39	**0.001**
concentration [32 μg/mL]	-25.90	-39.80 – -11.99	**<0.001**
concentration [64 μg/mL]	-20.78	-34.69 – -6.87	**0.004**
concentration [128 μg/mL]	-18.37	-32.28 – -4.46	**0.011**
$N_{\text{Replicate Culture}}$	3		
Observations	60		

Supplementary Table S15. Percent live model output for mature *M. bovis* PG45 biofilms exposed to tetracycline.

	Live Percent		
Predictors	*Estimates*	*CI*	*p*
(intercept) concentration [0 µg/mL]	83.77	69.12 – 98.42	**<0.001**
concentration [0.0625 µg/mL]	-11.19	-25.04 – 2.66	0.111
concentration [0.125 µg/mL]	-3.47	-17.33 – 10.38	0.616
concentration [0.25 µg/mL]	-18.41	-32.26 – -4.56	**0.010**
concentration [0.5 µg/mL]	-12.28	-26.13 – 1.57	0.081
concentration [1 µg/mL]	-12.57	-26.42 – 1.28	0.074
concentration [2 µg/mL]	-16.57	-30.43 – -2.72	**0.020**
concentration [4 µg/mL]	-10.93	-24.78 – 2.93	0.119
concentration [8 µg/mL]	-11.82	-25.68 – 2.03	0.093
concentration [16 µg/mL]	-1.82	-15.67 – 12.04	0.793
$N_{Replicate\ Culture}$	3		
Observations	60		

Supplementary Table S16. Percent live model output for newly adhered *M. bovis* PG45 biofilms exposed to enrofloxacin.

	Live Percent		
Predictors	*Estimates*	*CI*	*p*
(intercept) concentration [0 µg/mL]	92.01	89.71 – 94.31	**<0.001**
concentration [0.039 µg/mL]	-1.66	-4.45 – 1.12	0.236
concentration [0.078 µg/mL]	-2.63	-5.41 – 0.16	0.064
concentration [0.156 µg/mL]	-2.40	-5.19 – 0.38	0.089
concentration [0.312 µg/mL]	-0.31	-3.09 – 2.48	0.826
concentration [0.625 µg/mL]	-0.69	-3.47 – 2.10	0.623
concentration [1.35 µg/mL]	-0.47	-3.26 – 2.32	0.735
concentration [2.5 µg/mL]	-0.14	-2.93 – 2.65	0.921
concentration [5 µg/mL]	-0.48	-3.27 – 2.31	0.732
concentration [10 µg/mL]	-2.21	-4.99 – 0.58	0.118
$N_{\text{Replicate Culture}}$	3		
Observations	60		

Supplementary Table S17. Percent live model output for newly adhered *M. bovis* PG45 biofilms exposed to gentamicin.

	Live Percent		
Predictors	*Estimates*	*CI*	*p*
(intercept) concentration [0 μg/mL]	92.53	88.59 – 96.47	**<0.001**
concentration [0.5 μg/mL]	0.49	-2.07 – 3.05	0.702
concentration [1 μg/mL]	0.69	-1.87 – 3.25	0.592
concentration [2 μg/mL]	-0.55	-3.11 – 2.01	0.668
concentration [4 μg/mL]	0.76	-1.80 – 3.32	0.555
concentration [8 μg/mL]	1.26	-1.30 – 3.82	0.326
concentration [16 μg/mL]	-0.06	-2.62 – 2.50	0.965
concentration [32 μg/mL]	-0.06	-2.62 – 2.50	0.961
concentration [64 μg/mL]	-0.89	-3.45 – 1.67	0.486
concentration [128 μg/mL]	0.10	-2.46 – 2.66	0.940
$N_{\text{Replicate Culture}}$	3		
Observations	60		

Supplementary Table S18. Percent live model output for newly adhered *M. bovis* PG45 biofilms exposed to tetracycline.

	Live Percent		
Predictors	*Estimates*	*CI*	*p*
(intercept) concentration [0 µg/mL]	93.37	90.68 – 96.06	**<0.001**
concentration [0.0625 µg/mL]	-4.95	-8.06 – -1.84	**0.002**
concentration [0.125 µg/mL]	0.90	-2.21 – 4.01	0.564
concentration [0.25 µg/mL]	1.97	-1.14 – 5.08	0.208
concentration [0.5 µg/mL]	0.53	-2.58 – 3.63	0.736
concentration [1 µg/mL]	0.42	-2.69 – 3.53	0.788
concentration [2 µg/mL]	-0.13	-3.24 – 2.98	0.935
concentration [4 µg/mL]	-0.39	-3.50 – 2.72	0.801
concentration [8 µg/mL]	-0.50	-3.60 – 2.61	0.750
concentration [16 µg/mL]	-0.67	-3.78 – 2.43	0.664
$N_{\text{Replicate Culture}}$	3		
Observations	60		

Supplementary Table S19. Percent live model output for planktonic *M. bovis* PG45 cells exposed to enrofloxacin.

	Live Percent		
Predictors	*Estimates*	*CI*	*p*
(intercept) concentration [0 μg/mL]	88.03	74.48 – 101.58	**<0.001**
concentration [0.039 μg/mL]	-3.60	-13.39 – 6.18	0.463
concentration [0.078 μg/mL]	-6.62	-16.41 – 3.17	0.180
concentration [0.156 μg/mL]	-14.76	-24.55 – -4.97	**0.004**
concentration [0.312 μg/mL]	-20.35	-30.14 – -10.57	**<0.001**
concentration [0.625 μg/mL]	-18.05	-27.84 – -8.26	**0.001**
concentration [1.35 μg/mL]	-19.03	-28.82 – -9.24	**<0.001**
concentration [2.5 μg/mL]	-25.87	-35.66 – -16.08	**<0.001**
concentration [5 μg/mL]	-24.11	-33.90 – -14.32	**<0.001**
concentration [10 μg/mL]	-29.37	-39.15 – -19.58	**<0.001**
$N_{Replicate\ Culture}$	3		
Observations	60		

Supplementary Table S20. Percent live model output for planktonic *M. bovis* PG45 cells exposed to gentamicin.

	Live Percent		
Predictors	*Estimates*	*CI*	*p*
(intercept) concentration [0 µg/mL]	90.94	84.69 – 97.19	**<0.001**
concentration [0.5 µg/mL]	-2.99	-8.44 – 2.46	0.275
concentration [1 µg/mL]	-4.79	-10.23 – 0.66	0.084
concentration [2 µg/mL]	-4.28	-9.73 – 1.17	0.121
concentration [4 µg/mL]	-8.94	-14.38 – -3.49	**0.002**
concentration [8 µg/mL]	-10.18	-15.63 – -4.73	**<0.001**
concentration [16 µg/mL]	-19.60	-25.05 – -14.15	**<0.001**
concentration [32 µg/mL]	-20.76	-26.21 – -15.31	**<0.001**
concentration [64 µg/mL]	-15.77	-21.22 – -10.32	**<0.001**
concentration [128 µg/mL]	-12.30	-17.75 – -6.85	**<0.001**
$N_{\text{Replicate Culture}}$	3		
Observations	60		

Supplementary Table S21. Percent live model output for planktonic *M. bovis* PG45 cells exposed to tetracycline.

	Live Percent		
Predictors	*Estimates*	*CI*	*p*
(intercept) concentration [0 μg/mL]	88.48	76.13 – 100.82	**<0.001**
concentration [0.0625 μg/mL]	-1.28	-9.88 – 7.33	0.767
concentration [0.125 μg/mL]	-15.81	-24.41 – -7.21	**0.001**
concentration [0.25 μg/mL]	-8.81	-17.42 – -0.21	**0.045**
concentration [0.5 μg/mL]	-18.68	-27.28 – -10.08	**<0.001**
concentration [1 μg/mL]	-17.44	-26.04 – -8.83	**<0.001**
concentration [2 μg/mL]	-19.98	-28.58 – -11.38	**<0.001**
concentration [4 μg/mL]	-19.57	-28.18 – -10.97	**<0.001**
concentration [8 μg/mL]	-16.70	-25.30 – -8.10	**<0.001**
concentration [16 μg/mL]	-19.02	-27.62 – -10.42	**<0.001**
$N_{Replicate\ Culture}$	3		
Observations	60		

Supplementary Table S22. Percent live model output for mature *M. bovis* Madison biofilms exposed to enrofloxacin.

	Live Percent		
Predictors	*Estimates*	*CI*	*p*
(Intercept) concentration [0 µg/mL]	72.49	48.78 – 96.21	**<0.001**
concentration [0.039 µg/mL]	-0.52	-8.64 – 7.60	0.897
concentration [0.078 µg/mL]	-8.53	-16.65 – -0.41	**0.040**
concentration [0.156 µg/mL]	-14.58	-22.70 – -6.46	**0.001**
concentration [0.312 µg/mL]	-13.48	-21.60 – -5.36	**0.002**
concentration [0.625 µg/mL]	-15.13	-23.25 – -7.01	**0.001**
concentration [1.25 µg/mL]	-15.85	-23.97 – -7.73	**<0.001**
concentration [2.5 µg/mL]	-12.42	-20.54 – -4.30	**0.004**
concentration [5 µg/mL]	-14.28	-22.40 – -6.16	**0.001**
concentration [10 µg/mL]	-9.84	-17.96 – -1.72	**0.019**
N_{BR}	2		
Observations	40		

Supplementary Table S23. Percent live model output for mature *M. bovis* Madison biofilms exposed to gentamicin

	Live Percent		
Predictors	*Estimates*	*CI*	*p*
(Intercept) concentration [0 µg/mL]	77.15	57.57 – 96.72	**<0.001**
concentration [0.5 µg/mL]	-5.39	-15.85 – 5.07	0.301
concentration [1 µg/mL]	-6.62	-17.09 – 3.84	0.206
concentration [2 µg/mL]	-12.64	-23.10 – -2.17	**0.020**
concentration [4 µg/mL]	-9.79	-20.25 – 0.67	0.066
concentration [8 µg/mL]	-19.81	-30.27 – -9.35	**0.001**
concentration [16 µg/mL]	-17.38	-27.85 – -6.92	**0.002**
concentration [32 µg/mL]	-24.09	-34.55 – -13.63	**<0.001**
concentration [64 µg/mL]	-22.83	-33.29 – -12.37	**<0.001**
concentration [128 µg/mL]	-23.38	-33.84 – -12.92	**<0.001**
N_{BR}	2		
Observations	40		

Supplementary Table S24. Percent live model output for mature *M. bovis* Madison biofilms exposed to tetracycline

	Live Percent		
Predictors	*Estimates*	*CI*	*p*
(Intercept) concentration [0 µg/mL]	70.28	46.05 – 94.52	**<0.001**
concentration [0.0625 µg/mL]	-11.62	-18.34 – -4.91	**0.001**
concentration [0.125 µg/mL]	-13.93	-20.64 – -7.21	**<0.001**
concentration [0.25 µg/mL]	-9.17	-15.89 – -2.46	**0.009**
concentration [0.5 µg/mL]	-8.72	-15.44 – -2.01	**0.013**
concentration [1 µg/mL]	-13.70	-20.42 – -6.99	**<0.001**
concentration [2 µg/mL]	-12.52	-19.24 – -5.81	**0.001**
concentration [4 µg/mL]	-8.18	-14.90 – -1.47	**0.019**
concentration [8 µg/mL]	-13.80	-20.52 – -7.09	**<0.001**
concentration [16 µg/mL]	-4.54	-11.26 – 2.17	0.177
N_{BR}	2		
Observations	40		

Supplementary Table S25. Percent live model output for newly adhered *M. bovis* Madison biofilms exposed to enrofloxacin

	Live Percent		
Predictors	*Estimates*	*CI*	*p*
(Intercept) concentration [0 μg/mL]	80.61	42.59 – 118.62	**<0.001**
concentration [0.039 μg/mL]	-2.75	-18.13 – 12.64	0.718
concentration [0.078 μg/mL]	-17.59	-32.97 – -2.20	**0.027**
concentration [0.156 μg/mL]	-38.45	-53.83 – -23.06	**<0.001**
concentration [0.312 μg/mL]	-37.09	-52.47 – -21.70	**<0.001**
concentration [0.625 μg/mL]	-37.60	-52.99 – -22.21	**<0.001**
concentration [1.25 μg/mL]	-36.85	-52.24 – -21.46	**<0.001**
concentration [2.5 μg/mL]	-40.86	-56.24 – -25.47	**<0.001**
concentration [5 μg/mL]	-35.96	-51.34 – -20.57	**<0.001**
concentration [10 μg/mL]	-40.92	-56.31 – -25.54	**<0.001**
N_{BR}	2		
Observations	40		

Supplementary Table S26. Percent live model output for newly adhered *M. bovis* Madison biofilms exposed to gentamicin

	Live Percent		
Predictors	*Estimates*	*CI*	*p*
(Intercept) concentration [0 µg/mL]	79.75	48.51 – 110.98	**<0.001**
concentration [0.5 µg/mL]	-0.74	-12.74 – 11.27	0.901
concentration [1 µg/mL]	-0.28	-12.29 – 11.73	0.963
concentration [2 µg/mL]	-2.73	-14.74 – 9.28	0.646
concentration [4 µg/mL]	-8.36	-20.37 – 3.65	0.165
concentration [8 µg/mL]	-7.56	-19.57 – 4.45	0.208
concentration [16 µg/mL]	-17.21	-29.22 – -5.20	**0.007**
concentration [32 µg/mL]	-34.67	-46.68 – -22.66	**<0.001**
concentration [64 µg/mL]	-40.39	-52.40 – -28.38	**<0.001**
concentration [128 µg/mL]	-39.31	-51.32 – -27.30	**<0.001**
N_{BR}	2		
Observations	40		

Supplementary Table S27. Percent live model output for newly adhered *M. bovis* Madison biofilms exposed to tetracycline

	Live Percent		
Predictors	*Estimates*	*CI*	*p*
(Intercept) concentration [0 μg/mL]	77.50	34.84 – 120.16	**0.001**
concentration [0.0625 μg/mL]	-20.53	-29.23 – -11.83	**<0.001**
concentration [0.125 μg/mL]	-30.65	-39.34 – -21.95	**<0.001**
concentration [0.25 μg/mL]	-35.10	-43.80 – -26.41	**<0.001**
concentration [0.5 μg/mL]	-33.15	-41.84 – -24.45	**<0.001**
concentration [1 μg/mL]	-34.47	-43.17 – -25.78	**<0.001**
concentration [2 μg/mL]	-34.84	-43.54 – -26.15	**<0.001**
concentration [4 μg/mL]	-35.78	-44.47 – -27.08	**<0.001**
concentration [8 μg/mL]	-34.68	-43.38 – -25.98	**<0.001**
concentration [16 μg/mL]	-37.83	-46.53 – -29.14	**<0.001**
N_{BR}	2		
Observations	40		

Supplementary Table S28. Percent live model output for planktonic *M. bovis* Madison exposed to enrofloxacin

	Live Percent		
Predictors	*Estimates*	*CI*	*p*
(Intercept) concentration [0 μg/mL]	78.69	69.01 – 88.38	**<0.001**
concentration [0.039 μg/mL]	-1.72	-13.56 – 10.12	0.768
concentration [0.078 μg/mL]	-17.12	-28.96 – -5.28	**0.006**
concentration [0.156 μg/mL]	-12.88	-24.72 – -1.04	**0.034**
concentration [0.312 μg/mL]	-14.01	-25.85 – -2.17	**0.022**
concentration [0.625 μg/mL]	-17.81	-29.65 – -5.97	**0.005**
concentration [1.25 μg/mL]	-16.31	-28.15 – -4.47	**0.009**
concentration [2.5 μg/mL]	-17.61	-29.45 – -5.77	**0.005**
concentration [5 μg/mL]	-19.83	-31.67 – -7.99	**0.002**
concentration [10 μg/mL]	-28.32	-40.16 – -16.48	**<0.001**
N_{BR}	2		
Observations	40		

Supplementary Table S29. Percent live model output for planktonic *M. bovis* Madison exposed to gentamicin

	Live Percent		
Predictors	*Estimates*	*CI*	*p*
(Intercept) concentration [0 µg/mL]	76.33	62.20 – 90.45	**<0.001**
concentration [0.5 µg/mL]	0.21	-13.75 – 14.18	0.975
concentration [1 µg/mL]	2.11	-11.86 – 16.08	0.760
concentration [2 µg/mL]	-3.46	-17.43 – 10.51	0.617
concentration [4 µg/mL]	-13.67	-27.64 – 0.30	0.055
concentration [8 µg/mL]	-23.50	-37.47 – -9.53	**0.002**
concentration [16 µg/mL]	-22.05	-36.02 – -8.08	**0.003**
concentration [32 µg/mL]	-23.25	-37.22 – -9.29	**0.002**
concentration [64 µg/mL]	-35.95	-49.92 – -21.98	**<0.001**
concentration [128 µg/mL]	-31.37	-45.34 – -17.41	**<0.001**
N_{BR}	2		
Observations	40		

Supplementary Table S30. Percent live model output for planktonic *M. bovis* Madison exposed to tetracycline

	Live Percent		
Predictors	*Estimates*	*CI*	*p*
(Intercept) concentration [0 µg/mL]	77.96	71.05 – 84.87	**<0.001**
concentration [0.0625 µg/mL]	-0.43	-10.20 – 9.34	0.929
concentration [0.125 µg/mL]	-10.58	-20.34 – -0.81	**0.035**
concentration [0.25 µg/mL]	-15.36	-25.13 – -5.59	**0.003**
concentration [0.5 µg/mL]	-13.13	-22.90 – -3.37	**0.010**
concentration [1 µg/mL]	-17.07	-26.84 – -7.30	**0.001**
concentration [2 µg/mL]	-15.30	-25.07 – -5.54	**0.003**
concentration [4 µg/mL]	-15.75	-25.51 – -5.98	**0.003**
concentration [8 µg/mL]	-19.27	-29.04 – -9.50	**<0.001**
concentration [16 µg/mL]	-27.69	-37.46 – -17.92	**<0.001**
N_{BR}	2		
Observations	40		

Supplementary Table S31. Percent live model output for Mature *Mycoplasma sp.* MVDL1 biofilm exposed to enrofloxacin

	Live Percent		
Predictors	*Estimates*	*CI*	*p*
(Intercept) concentration [0 µg/mL]	90.93	67.67 – 114.18	**<0.001**
concentration [0.039 µg/mL]	-0.72	-20.13 – 18.69	0.940
concentration [0.078 µg/mL]	-10.71	-30.12 – 8.70	0.268
concentration [0.156 µg/mL]	-18.39	-37.80 – 1.02	0.062
concentration [0.312 µg/mL]	-19.78	-39.19 – -0.37	**0.046**
concentration [0.625 µg/mL]	-28.38	-47.78 – -8.97	**0.006**
concentration [1.25 µg/mL]	-25.00	-44.41 – -5.59	**0.013**
concentration [2.5 µg/mL]	-4.94	-24.35 – 14.47	0.606
concentration [5 µg/mL]	-3.69	-23.10 – 15.72	0.700
concentration [10 µg/mL]	-2.34	-21.74 – 17.07	0.807
N_{BR}	2		
Observations	40		

Supplementary Table S32. Percent live model output for Mature *Mycoplasma sp.* MVDL1 biofilm exposed to gentamicin

	Percent Live		
Predictors	*Estimates*	*CI*	*p*
(Intercept) concentration [0 µg/mL]	91.40	85.28 – 97.52	**<0.001**
concentration [0.5 µg/mL]	-3.08	-9.46 – 3.29	0.330
concentration [1 µg/mL]	-1.46	-7.83 – 4.91	0.643
concentration [2 µg/mL]	-2.51	-8.88 – 3.86	0.427
concentration [4 µg/mL]	-3.10	-9.47 – 3.27	0.327
concentration [8 µg/mL]	-4.86	-11.23 – 1.51	0.130
concentration [16 µg/mL]	-8.53	-14.90 – -2.16	**0.010**
concentration [32 µg/mL]	-9.58	-15.95 – -3.21	**0.005**
concentration [64 µg/mL]	-11.12	-17.49 – -4.75	**0.001**
concentration [128 µg/mL]	-9.08	-15.45 – -2.71	**0.007**
N_{BR}	2		
Observations	40		

Supplementary Table S33. Percent live model output for Mature *Mycoplasma sp.* MVDL1 biofilm exposed to tetracycline

	Live Percent		
Predictors	*Estimates*	*CI*	*p*
(Intercept) concentration [0 μg/mL]	92.86	86.76 – 98.97	**<0.001**
concentration [0.0625 μg/mL]	-2.11	-10.74 – 6.52	0.621
concentration [0.125 μg/mL]	-2.47	-11.10 – 6.16	0.563
concentration [0.25 μg/mL]	-2.62	-11.25 – 6.02	0.540
concentration [0.5 μg/mL]	-11.74	-20.38 – -3.11	**0.009**
concentration [1 μg/mL]	-2.93	-11.57 – 5.70	0.492
concentration [2 μg/mL]	-4.62	-13.25 – 4.01	0.283
concentration [4 μg/mL]	-4.22	-12.86 – 4.41	0.325
concentration [8 μg/mL]	-3.70	-12.33 – 4.93	0.388
concentration [16 μg/mL]	-6.71	-15.35 – 1.92	0.123
N_{BR}	2		
Observations	40		

Supplementary Table S34. Percent live model output for newly adhered *Mycoplasma sp.* MVDL1 biofilm exposed to enrofloxacin

	Live Percent		
Predictors	*Estimates*	*CI*	*p*
(Intercept) concentration [0 μg/mL]	88.09	79.86 – 96.33	**<0.001**
concentration [0.039 μg/mL]	3.30	-0.53 – 7.13	0.088
concentration [0.078 μg/mL]	4.95	1.12 – 8.78	**0.013**
concentration [0.156 μg/mL]	5.21	1.38 – 9.04	**0.009**
concentration [0.312 μg/mL]	4.99	1.16 – 8.82	**0.012**
concentration [0.625 μg/mL]	5.44	1.61 – 9.27	**0.007**
concentration [1.25 μg/mL]	4.16	0.33 – 7.99	**0.034**
concentration [2.5 μg/mL]	4.89	1.06 – 8.72	**0.014**
concentration [5 μg/mL]	5.20	1.37 – 9.03	**0.009**
concentration [10 μg/mL]	4.82	0.99 – 8.64	**0.015**
N_{BR}	2		
Observations	40		

Supplementary Table S35. Percent live model output for newly adhered *Mycoplasma sp.* MVDL1 biofilm exposed to gentamicin

	Live Percent		
Predictors	*Estimates*	*CI*	*p*
(Intercept) concentration [0 μg/mL]	80.65	71.76 – 89.53	**<0.001**
concentration [0.5 μg/mL]	3.70	-3.59 – 11.00	0.308
concentration [1 μg/mL]	3.89	-3.40 – 11.19	0.284
concentration [2 μg/mL]	10.92	3.62 – 18.21	**0.005**
concentration [4 μg/mL]	9.50	2.20 – 16.79	**0.013**
concentration [8 μg/mL]	9.49	2.19 – 16.78	**0.013**
concentration [16 μg/mL]	10.04	2.74 – 17.33	**0.009**
concentration [32 μg/mL]	11.08	3.79 – 18.38	**0.004**
concentration [64 μg/mL]	8.14	0.85 – 15.43	**0.030**
concentration [128 μg/mL]	9.93	2.64 – 17.23	**0.009**
N_{BR}	2		
Observations	40		

Supplementary Table S36. Percent live model output for newly adhered *Mycoplasma sp.* MVDL1 biofilm exposed to tetracycline

	Percent Live		
Predictors	*Estimates*	*CI*	*p*
(Intercept) concentration [0 µg/mL]	87.94	77.60 – 98.27	**<0.001**
concentration [0.0625 µg/mL]	2.97	-1.67 – 7.60	0.201
concentration [0.125 µg/mL]	5.20	0.57 – 9.83	**0.029**
concentration [0.25 µg/mL]	6.64	2.00 – 11.27	**0.007**
concentration [0.5 µg/mL]	6.88	2.25 – 11.51	**0.005**
concentration [1 µg/mL]	5.35	0.72 – 9.98	**0.025**
concentration [2 µg/mL]	5.08	0.45 – 9.72	**0.033**
concentration [4 µg/mL]	5.32	0.68 – 9.95	**0.026**
concentration [8 µg/mL]	5.28	0.64 – 9.91	**0.027**
concentration [16 µg/mL]	4.39	-0.24 – 9.03	0.062
N_{BR}	2		
Observations	40		

Supplementary Table S37. Percent live model output for planktonic *Mycoplasma sp.* MVDL1 exposed to enrofloxacin

	Live Percent		
Predictors	*Estimates*	*CI*	*p*
(Intercept) concentration [0 μg/mL]	93.83	83.10 – 104.55	**<0.001**
concentration [0.039 μg/mL]	-13.16	-20.21 – -6.10	**0.001**
concentration [0.078 μg/mL]	-14.36	-21.42 – -7.31	**<0.001**
concentration [0.156 μg/mL]	-22.27	-29.33 – -15.22	**<0.001**
concentration [0.312 μg/mL]	-21.12	-28.17 – -14.06	**<0.001**
concentration [0.625 μg/mL]	-24.09	-31.14 – -17.03	**<0.001**
concentration [1.25 μg/mL]	-24.20	-31.25 – -17.14	**<0.001**
concentration [2.5 μg/mL]	-24.55	-31.60 – -17.49	**<0.001**
concentration [5 μg/mL]	-21.48	-28.54 – -14.43	**<0.001**
concentration [10 μg/mL]	-22.87	-29.92 – -15.82	**<0.001**
N BR	2		
Observations	40		

Supplementary Table S38. Percent live model output for planktonic *Mycoplasma sp.* MVDL1 exposed to gentamicin

	Live Percent		
Predictors	*Estimates*	*CI*	*p*
(Intercept) concentration [0 μg/mL]	91.53	74.77 – 108.28	**<0.001**
concentration [0.5 μg/mL]	-13.94	-28.22 – 0.35	0.056
concentration [1 μg/mL]	-13.84	-28.13 – 0.44	0.057
concentration [2 μg/mL]	-19.77	-34.06 – -5.49	**0.008**
concentration [4 μg/mL]	-16.33	-30.62 – -2.05	**0.026**
concentration [8 μg/mL]	-26.50	-40.79 – -12.22	**0.001**
concentration [16 μg/mL]	-28.37	-42.66 – -14.09	**<0.001**
concentration [32 μg/mL]	-32.69	-46.97 – -18.40	**<0.001**
concentration [64 μg/mL]	-33.32	-47.60 – -19.03	**<0.001**
concentration [128 μg/mL]	-36.57	-50.86 – -22.29	**<0.001**
N_{BR}	2		
Observations	40		

Supplementary Table S39. Percent live model output for planktonic *Mycoplasma sp.* MVDL1 exposed to tetracycline

	Live Percent		
Predictors	*Estimates*	*CI*	*p*
(Intercept) concentration [0 μg/mL]	91.50	86.06 – 96.94	**<0.001**
concentration [0.0625 μg/mL]	-10.35	-17.77 – -2.93	**0.008**
concentration [0.125 μg/mL]	-20.04	-27.46 – -12.62	**<0.001**
concentration [0.25 μg/mL]	-23.86	-31.28 – -16.44	**<0.001**
concentration [0.5 μg/mL]	-19.43	-26.85 – -12.01	**<0.001**
concentration [1 μg/mL]	-20.99	-28.41 – -13.57	**<0.001**
concentration [2 μg/mL]	-18.76	-26.18 – -11.33	**<0.001**
concentration [4 μg/mL]	-21.86	-29.28 – -14.44	**<0.001**
concentration [8 μg/mL]	-23.70	-31.12 – -16.28	**<0.001**
concentration [16 μg/mL]	-18.54	-25.96 – -11.12	**<0.001**
N_{BR}	2		
Observations	40		

Supplementary Table S40. Percent live model output for mature *Mycoplasma sp.* MVDL2 biofilm exposed to enrofloxacin

		**Live Percent**	
Predictors	*Estimates*	*CI*	*p*
(Intercept) concentration [0 μg/mL]	90.09	68.78 – 111.40	**<0.001**
concentration [0.039 μg/mL]	-3.49	-10.30 – 3.32	0.303
concentration [0.078 μg/mL]	-7.06	-13.87 – -0.25	**0.043**
concentration [0.156 μg/mL]	-10.79	-17.60 – -3.98	**0.003**
concentration [0.312 μg/mL]	-12.09	-18.90 – -5.28	**0.001**
concentration [0.625 μg/mL]	-8.59	-15.40 – -1.78	**0.015**
concentration [1.25 μg/mL]	-10.70	-17.51 – -3.89	**0.003**
concentration [2.5 μg/mL]	-11.41	-18.22 – -4.60	**0.002**
concentration [5 μg/mL]	-13.28	-20.09 – -6.47	**<0.001**
concentration [10 μg/mL]	-10.24	-17.05 – -3.43	**0.005**
N_{BR}	2		
Observations	40		

Supplementary Table S41. Percent live model output for mature *Mycoplasma sp.* MVDL2 biofilm exposed to gentamicin

	Live Percent		
Predictors	*Estimates*	*CI*	*p*
(Intercept) concentration [0 µg/mL]	88.83	69.42 – 108.23	**<0.001**
concentration [0.5 µg/mL]	1.62	-5.28 – 8.52	0.635
concentration [1 µg/mL]	-2.55	-9.45 – 4.36	0.457
concentration [2 µg/mL]	-4.59	-11.49 – 2.31	0.184
concentration [4 µg/mL]	-9.32	-16.23 – -2.42	**0.010**
concentration [8 µg/mL]	-10.22	-17.12 – -3.31	**0.005**
concentration [16 µg/mL]	-12.18	-19.08 – -5.27	**0.001**
concentration [32 µg/mL]	-14.88	-21.78 – -7.98	**<0.001**
concentration [64 µg/mL]	-14.81	-21.71 – -7.91	**<0.001**
concentration [128 µg/mL]	-8.97	-15.87 – -2.06	**0.013**
N_{BR}	2		
Observations	40		

Supplementary Table S42. Percent live model output for mature *Mycoplasma sp.* MVDL2 biofilm exposed to tetracycline

	Dependent variable		
Predictors	*Estimates*	*CI*	*p*
(Intercept) concentration [0 μg/mL]	86.99	68.58 – 105.40	**<0.001**
concentration [0.0625 μg/mL]	-8.40	-15.76 – -1.04	**0.027**
concentration [0.125 μg/mL]	-7.64	-15.00 – -0.28	**0.042**
concentration [0.25 μg/mL]	-11.66	-19.02 – -4.30	**0.003**
concentration [0.5 μg/mL]	-10.58	-17.94 – -3.22	**0.006**
concentration [1 μg/mL]	-8.91	-16.27 – -1.55	**0.019**
concentration [2 μg/mL]	-8.42	-15.78 – -1.06	**0.026**
concentration [4 μg/mL]	-6.44	-13.80 – 0.92	0.084
concentration [8 μg/mL]	-9.89	-17.25 – -2.53	**0.010**
concentration [16 μg/mL]	-1.53	-8.89 – 5.83	0.673
N_{BR}	2		
Observations	40		

Supplementary Table S43. Percent live model output for newly adhered *Mycoplasma sp.* MVDL2 biofilm exposed to enrofloxacin

	Live Percent		
Predictors	*Estimates*	*CI*	*p*
(Intercept) concentration [0 µg/mL]	81.32	75.19 – 87.44	**<0.001**
concentration [0.039 µg/mL]	3.26	-5.40 – 11.92	0.448
concentration [0.078 µg/mL]	15.63	6.97 – 24.29	**0.001**
concentration [0.156 µg/mL]	16.48	7.82 – 25.14	**0.001**
concentration [0.312 µg/mL]	16.13	7.47 – 24.79	**0.001**
concentration [0.625 µg/mL]	16.33	7.67 – 25.00	**0.001**
concentration [1.25 µg/mL]	16.43	7.77 – 25.09	**0.001**
concentration [2.5 µg/mL]	16.26	7.60 – 24.92	**0.001**
concentration [5 µg/mL]	16.74	8.08 – 25.40	**<0.001**
concentration [10 µg/mL]	16.77	8.11 – 25.43	**<0.001**
N_{BR}	2		
Observations	40		

Supplementary Table S44. Percent live model output for newly adhered *Mycoplasma sp.* MVDL2 biofilm exposed to gentamicin

	Live Percent		
Predictors	*Estimates*	*CI*	*p*
(Intercept) concentration [0 μg/mL]	77.01	67.70 – 86.32	**<0.001**
concentration [0.5 μg/mL]	7.84	-5.33 – 21.02	0.233
concentration [1 μg/mL]	12.66	-0.51 – 25.84	0.059
concentration [2 μg/mL]	8.92	-4.25 – 22.09	0.177
concentration [4 μg/mL]	18.45	5.28 – 31.62	**0.008**
concentration [8 μg/mL]	18.70	5.52 – 31.87	**0.007**
concentration [16 μg/mL]	17.86	4.68 – 31.03	**0.010**
concentration [32 μg/mL]	18.67	5.49 – 31.84	**0.007**
concentration [64 μg/mL]	19.09	5.91 – 32.26	**0.006**
concentration [128 μg/mL]	18.76	5.59 – 31.94	**0.007**
N_{BR}	2		
Observations	40		

Supplementary Table S45. Percent live model output for newly adhered *Mycoplasma sp.* MVDL2 biofilm exposed to tetracycline

	Live Percent		
Predictors	*Estimates*	*CI*	*p*
(Intercept) concentration [0 μg/mL]	90.15	86.87 – 93.44	**<0.001**
concentration [0.0625 μg/mL]	6.82	2.57 – 11.07	**0.003**
concentration [0.125 μg/mL]	7.24	2.99 – 11.48	**0.002**
concentration [0.25 μg/mL]	7.49	3.24 – 11.74	**0.001**
concentration [0.5 μg/mL]	8.14	3.89 – 12.39	**<0.001**
concentration [1 μg/mL]	7.79	3.54 – 12.03	**0.001**
concentration [2 μg/mL]	7.80	3.55 – 12.05	**0.001**
concentration [4 μg/mL]	7.80	3.55 – 12.04	**0.001**
concentration [8 μg/mL]	7.41	3.16 – 11.66	**0.001**
concentration [16 μg/mL]	7.58	3.33 – 11.82	**0.001**
N_{BR}	2		
Observations	40		

Supplementary Table S46. Percent live model output for planktonic *Mycoplasma sp.* MVDL2 exposed to enrofloxacin

	Live Percent		
Predictors	*Estimates*	*CI*	*p*
(Intercept) concentration [0 µg/mL]	70.23	65.96 – 74.49	**<0.001**
concentration [0.039 µg/mL]	-0.66	-6.55 – 5.24	0.821
concentration [0.078 µg/mL]	-6.19	-12.09 – -0.30	**0.040**
concentration [0.156 µg/mL]	-10.26	-16.15 – -4.36	**0.001**
concentration [0.312 µg/mL]	-6.96	-12.85 – -1.06	**0.022**
concentration [0.625 µg/mL]	-7.09	-12.99 – -1.20	**0.020**
concentration [1.25 µg/mL]	-7.38	-13.28 – -1.48	**0.016**
concentration [2.5 µg/mL]	-5.47	-11.37 – 0.43	0.068
concentration [5 µg/mL]	-7.67	-13.56 – -1.77	**0.013**
concentration [10 µg/mL]	-9.06	-14.96 – -3.16	**0.004**
N_{BR}	2		
Observations	40		

Supplementary Table S47. Percent live model output for planktonic *Mycoplasma sp.* MVDL2 exposed to gentamicin

	Live Percent		
Predictors	*Estimates*	*CI*	*p*
(Intercept) concentration [0 μg/mL]	73.43	65.54 – 81.32	**<0.001**
concentration [0.5 μg/mL]	-1.17	-12.33 – 9.98	0.831
concentration [1 μg/mL]	-1.44	-12.60 – 9.71	0.793
concentration [2 μg/mL]	-5.92	-17.07 – 5.24	0.287
concentration [4 μg/mL]	-5.86	-17.01 – 5.29	0.291
concentration [8 μg/mL]	-3.17	-14.32 – 7.98	0.565
concentration [16 μg/mL]	-11.55	-22.70 – -0.39	**0.043**
concentration [32 μg/mL]	-11.74	-22.90 – -0.59	**0.040**
concentration [64 μg/mL]	-14.99	-26.15 – -3.84	**0.010**
concentration [128 μg/mL]	-17.31	-28.46 – -6.15	**0.004**
N_{BR}	2		
Observations	40		

Supplementary Table S48. Percent live model output for planktonic *Mycoplasma sp.* MVDL2 exposed to tetracycline

	Live Percent		
Predictors	*Estimates*	*CI*	*p*
(Intercept) concentration [0 μg/mL]	75.99	71.72 – 80.26	**<0.001**
concentration [0.0625 μg/mL]	-5.67	-11.72 – 0.37	0.065
concentration [0.125 μg/mL]	-11.12	-17.16 – -5.08	**0.001**
concentration [0.25 μg/mL]	-16.72	-22.76 – -10.68	**<0.001**
concentration [0.5 μg/mL]	-16.23	-22.27 – -10.18	**<0.001**
concentration [1 μg/mL]	-10.34	-16.39 – -4.30	**0.002**
concentration [2 μg/mL]	-13.14	-19.19 – -7.10	**<0.001**
concentration [4 μg/mL]	-11.96	-18.00 – -5.92	**<0.001**
concentration [8 μg/mL]	-11.46	-17.50 – -5.42	**0.001**
concentration [16 μg/mL]	-17.72	-23.76 – -11.67	**<0.001**
N_{BR}	2		
Observations	40		
